# Supplementary material for: Data-driven RNA phenotyping captures genetically regulated dimensions of the transcriptome
Source: Am J Hum Genet. 2026 Jul 2;113(7):1399–414. doi: 10.1016/j.ajhg.2026.06.005 (PMC13361010; doi:10.1016/j.ajhg.2026.06.005)
Supplement: Supplementary file 2 [file mmc5.pdf]

Data-driven RNA phenotyping captures genetically regulated dimensions of the transcriptome

Authors

Daniel Munro, Alexander Gusev,
Abraham A. Palmer, Pejman Mohammadi

Correspondence

pejmanm@uw.edu

Genetic variants can regulate many aspects of transcription, any of which could influence complex traits. We developed LaDDR, a method for generating latent data-driven RNA phenotypes from RNA sequencing read coverage. LaDDR substantially increases discovery of regulatory variation and gene-trait associations and can be integrated with knowledge-driven RNA phenotypes.

Munro et al., 2026, The American Journal of Human Genetics *113*, 1399–1414
July 2, 2026 © 2026 The Author(s). Published by Elsevier Inc. on behalf of American Society of Human Genetics.
https://doi.org/10.1016/j.ajhg.2026.06.005

Data-driven RNA phenotyping captures genetically regulated dimensions of the transcriptome

Daniel Munro,[1,2] Alexander Gusev,[3] Abraham A. Palmer,[1,4] and Pejman Mohammadi[2,5,*]

Summary

Transcriptomic diversity across individuals arises from multiple modes of RNA regulation—including precursor messenger RNA (pre-mRNA) expression, splicing, degradation, and other processes—and has been widely leveraged to map molecular quantitative trait loci (xQTLs) and interpret genome-wide association study (GWAS) signals. We recently developed a multimodal framework called Pantry that can extend discovery beyond total expression by integrating multiple transcriptomic modalities. However, Pantry and similar tools remain limited by their reliance on complete gene annotations and the statistical complexity of jointly analyzing correlated modalities. Here, we present LaDDR (latent data-driven RNA phenotyping), a mechanism-agnostic framework that generates orthogonal, latent coverage features per gene, enabling xQTL discovery and GWAS integration without requiring complete gene annotations. Applied to the Genotype-Tissue Expression (GTEx) Project, LaDDR identified an average of 95% more independent xQTLs per tissue than the six transcriptional regulation modes implemented in Pantry ("knowledge-driven"). Residualizing known modalities prior to LaDDR and combining with knowledge-driven phenotypes increased discovery by an additional 41% per tissue on average while retaining the interpretability of knowledge-driven signals. In a transcriptome-wide association study (TWAS) of 114 complex traits, the use of LaDDR-derived phenotypes uncovered an average of 11,796 unique gene-trait pairs per tissue versus 8,630 from knowledge-driven phenotypes. The newly captured genetic signals exhibit functional and colocalization qualities consistent with known mechanisms, suggesting that LaDDR broadens the detectable landscape of trait-relevant transcriptomic regulation by efficiently recovering regulatory variation missed by current pipelines.

Introduction

Transcriptome sequencing data have been used to characterize variation in transcriptome regulation across individuals.[1–4] Integration with genome variation data has been used extensively to understand the regulatory mechanisms that mediate disease risk and variation in complex traits.[5–8] While these analyses were originally introduced using gene expression data, over the years several other modes of transcriptome regulation, such as splicing, the use of alternative transcript start and end sites, and others, have further expanded the scope of transcriptome analysis.[9–12] The conventional approach to transcriptome analysis involves building specific tools designed to quantify each specific mode of transcriptome regulation in an RNA sequencing (RNA-seq) library based on the mechanistic knowledge.

While this knowledge-driven phenotyping approach maximizes interpretability, it has several limitations. First, it relies on potentially inaccurate annotations of transcriptome products and regulatory events specific to each mode of regulation. This complicates phenotype extraction because unannotated regulatory events are either excluded or reported inappropriately under known annotation entries.[13] Notably, a study of 90 Genotype-Tissue Expression (GTEx) samples for which long-read sequencing data were available identified 70,000 unannotated transcript isoforms for annotated genes in the human genome.[14] Conversely, not all annotated transcript isoforms can be accurately quantified from short-read sequencing data. Another limitation of knowledge-driven phenotyping is the pervasive correlations within the data, which lead to false discoveries, redundant findings, and a loss of statistical power. These include biological correlations, technical artifacts in quantification, and systematic correlations across different modes of transcription regulation.[15]

Finally, incorporating multiple modes of transcriptome regulation into a single analysis is often too cumbersome due to practical constraints, including the need to harmonize and run many packages and the need for domain-specific expertise. Tools such as the eQTL-Catalogue/rnaseq pipeline[16] and Pantry[17] aim to alleviate this issue by curating and harmonizing different transcriptome phenotyping software to extract multiple phenotypic modalities from RNA-seq data. Pantry currently quantifies 6 modalities: expression, isoform ratio, intron excision ratio, alternative transcription start site (TSS), alternative poly(A), and RNA stability.

Data-driven phenotyping approaches can address all three of these limitations by efficiently capturing variation in population transcriptome data without relying on detailed annotations. Other annotation-agnostic methods have been introduced previously to address

[1]Department of Psychiatry, UC San Diego, La Jolla, CA, USA; [2]Center for Immunity and Immunotherapies, Seattle Children's Research Institute, Seattle, WA, USA; [3]Division of Population Sciences, Dana-Farber Cancer Institute and Harvard Medical School, Boston, MA, USA; [4]Institute for Genomic Medicine, UC San Diego, La Jolla, CA, USA; [5]Department of Pediatrics, University of Washington School of Medicine, Seattle, WA, USA
*Correspondence: pejmanm@uw.edu
https://doi.org/10.1016/j.ajhg.2026.06.005.
© 2026 The Author(s). Published by Elsevier Inc. on behalf of American Society of Human Genetics.
This is an open access article under the CC BY license (http://creativecommons.org/licenses/by/4.0/).

this challenge for certain use cases. For example, SCISSOR uses base-level RNA-seq coverage to detect outliers in read depth within each gene.[18] Another method, derfinder, identifies base-level differential expression from RNA-seq coverage data.[19,20]

Here, we introduce LaDDR (latent data-driven RNA phenotyping), which extracts latent orthogonal dimensions of variation for each gene, serving as transcriptional phenotypes from RNA-seq data, thereby increasing its utility for QTL analysis and genome-wide association study (GWAS) integration. We show that LaDDR's data-driven phenotypes (DDPs) yield more molecular quantitative trait locus (xQTL) and transcriptome-wide association study (TWAS) associations than Pantry's six knowledge-driven modalities combined. Furthermore, we show that LaDDR can be used in conjunction with conventional phenotyping tools to provide even greater discovery power while retaining data interpretability. We provide a software package, pre-built models for 49 human tissues, and data including DDPs and results of their application to genetic analyses.

Methods

LaDDR phenotyping

The input data are base-level RNA-seq coverage files in bigWig format. Protein-coding and long non-coding RNA (lncRNA) genes are first partitioned into bins using an adaptive binning process. RNA-seq coverage at every base across the gene, from start to end plus 1 kb upstream and downstream, is extracted for 256 randomly chosen samples from the training set. Values are $\log_2$ transformed after adding a pseudocount (eight by default), the coverage difference between consecutive bases is computed, and the variance of these differences is computed for each base. Starting at the beginning of the region, bases are included in the current bin until the cumulative sum of variance differences reaches a predefined threshold, after which the next bin begins. This results in a higher density of smaller bins in regions of the gene with more transcriptional variation. The threshold, which is fixed across all genes, is determined from a random sample of 128 genes and is calculated to produce a desired average number of bins per gene (256 by default). Bins larger than 1,024 bases are subdivided uniformly such that the maximum bin size is 1,024. Finally, any bin overlapping an exonic region of a different gene is removed to reduce contamination of a gene's RNA-seq coverage by reads from a different gene's transcript.

Using the bin definitions, base-level coverage counts are summarized into mean coverage per bin per sample using pyBigWig.[21] A scaling factor is computed for each sample based on cumulative coverage level relative to the median sample level, and coverage values are divided by the scaling factors to adjust for sequencing depths. A pseudocount (eight by default) is added to the coverage values, which are then $\log_2$ transformed to stabilize variance.

At this stage, knowledge-driven phenotypes, such as those produced by Pantry, can be regressed out of the normalized binned coverage features to represent residual transcriptomic variation. This occurs on a per-gene basis, with the following procedure applied to each bin feature using all knowledge-driven phenotypes (KDPs) for the same gene. First, an ordinary least squares linear regression is fit using all of the gene's KDPs as features and normalized coverage for the bin as the response variable. KDPs with regression $p < 0.01$ are kept, and linear regression is repeated with only those KDPs as variables. The regression model predictions on the same data are subtracted from the bin feature, and the residuals are used in place of the original coverage features in subsequent steps.

The normalized binned coverage data or the residualized data are then standardized and used to fit a principal-component analysis (PCA) model for each gene using the scikit-learn Python package.[22] Genes are filtered such that models are only produced for those with nonzero total coverage in at least 50% of samples. While the number of principal components (PCs) is necessarily capped at the minimum of the number of bins and the number of samples, additional caps can be set to explain up to a desired percentage of variance and a maximum number of PCs. By default and for this study, enough PCs to explain 80% of variance, up to a maximum of 16, were used. Genes are processed in batches for efficiency, and data can be concatenated across multiple datasets to generate models representing diverse biological contexts.

These PCA models are then applied to the same normalized, binned coverage data and/or new data, one dataset at a time, and the resulting PC projections are the DDPs or residual DDPs (rDDPs). Genes are again filtered for nonzero total coverage in at least 50% of samples, this time in the set of samples for which phenotypes are being generated.

RNA-seq datasets

We downloaded all bigWig files for the 54 tissues from the GTEx Project (18,948 samples) and 33 cancer types from The Cancer Genome Atlas (TCGA; 11,287 samples) from recount3.[23] We subsetted the GTEx samples to those 17,350 included in the v.8 release to have at most one sample per individual per tissue. We also used RNA-seq data for 445 human lymphoblastoid cell line (LCL) samples from Geuvadis.[2] To get bigWig coverage files for Geuvadis, we mapped RNA-seq reads to the GRCh38 reference genome using STAR v.2.7.11b[24] with the parameters specified in the Pantry pipeline and then converted each BAM file to bigWig files using bamCoverage from deeptools[25] with –binSize 1.

Pantry data

Pantry was used to quantify KDPs for comparison with data-driven RNA phenotypes and to generate residual data-driven RNA phenotypes. We ran Pantry on Geuvadis and 49 GTEx tissues using Ensembl v.113 gene annotations,[26] producing KDPs for six modalities: expression, isoform ratio, intron excision ratio, alternative TSS, alternative poly(A), and RNA stability. For some analyses, such as the pruned annotation comparison, Pantry was rerun, e.g., with different gene annotations, on particular tissue datasets, as described in pruned annotation analysis and method and parameter comparisons.

Application of LaDDR phenotyping

We fitted and applied LaDDR DDP models to the combined set of 54 GTEx tissues and 33 TCGA cancer types using default parameters described above, and unless otherwise specified, these phenotypes were used for all reported analyses of GTEx DDPs. Likewise, we fit and applied LaDDR rDDP models to the 49 GTEx tissues for which we had Pantry phenotypes. DDP and

rDDP models were also fitted and applied to the Geuvadis dataset. For comparisons of input data size and diversity, we fit DDP models to the combined set of 54 GTEx tissues, as well as a subset of five GTEx tissues (adipose - subcutaneous, artery - aorta, brain - frontal cortex [BA9], esophagus - gastroesophageal junction, and skin - sun exposed [lower leg]), chosen randomly from each of the five largest tissue site categories in terms of unique tissues.

xQTL mapping

We performed *cis*-QTL mapping on DDPs using tensorQTL,[27] following the default Pantry Pheast pipeline. This procedure uses a permutation test on the nominal associations for each gene to account for multiple testing across *cis*-window variants, followed by calculation of q values across genes to determine significant *cis*-QTLs at a 5% false discovery rate (FDR) threshold, and finally, stepwise regression to identify any additional conditionally independent *cis*-QTLs per gene. For the hybrid phenotype set xQTLs, rDDPs were added to the set of KDPs as a seventh modality, and cross-modality mapping was performed accordingly. For DDP xQTLs, DDPs were mapped as a single modality, i.e., mapping of conditionally independent *cis*-QTLs on phenotypes grouped by gene.

TWAS

We generated multimodal TWAS (xTWAS) predictive models for DDPs and rDDPs and tested xTWAS associations using FUSION[6] with default parameters, as described previously.[17] We tested associations for a collection of 114 GWAS traits.[7,28] We used a genome-wide p value threshold of 5×10^{-8} to determine significant xTWAS hits. We also performed xTWAS for KDPs generated by Pantry using the same procedure and a p value threshold of 5×10^{-8} to compare to the DDP xTWAS results and to combine with the rDDP xTWAS results. Colocalization tests as implemented in the coloc R package[29] were applied to each TWAS hit, and hits with COLOC.PP4 (posterior probability of shared causal variant) > 0.8 were considered colocalizing TWAS hits for the purposes of this study.

Pruned annotation analysis

Starting with the Ensembl human v.113 gene annotations, the isoform per gene that was present (ignoring the Ensembl ID version) in a curated list of canonical isoforms from UCSC (http://hgdownload.soe.ucsc.edu/goldenPath/hg38/database/knownCanonical.txt.gz) was labeled as canonical. To simulate sparser gene annotations, non-canonical isoforms were subsampled successively to retain 80%, 60%, 40%, 20%, and 0% of the original amount, with 0% leaving only the one canonical isoform per gene. Each GTF file was then used to generate DDPs with LaDDR and KDPs with Pantry for one GTEx tissue, brain - frontal cortex (BA9) ($n = 175$ genotyped samples). We mapped conditionally independent *cis*-QTLs for each set of phenotypes using tensorQTL and compared the numbers of *cis*-QTLs.

Read-length and -depth simulations

We used a random subsample of 100 Geuvadis samples for all read-length and -depth simulations. We used seqtk trimfq (https://github.com/lh3/seqtk) to truncate all (75 bp) reads in the paired-end FASTQ files to 50 bp. We used seqtk sample to subsample reads in each FASTQ file to 50% and 25% of the original number by using a fixed random seed so that read pairs were preserved within R1/R2 file pairs. We then aligned reads in each simulated dataset by using STAR, generated bigWig coverage files by using bamCoverage, ran LaDDR, and mapped *cis*-QTLs on the DDPs by using tensorQTL, all in the same way as described above.

Method and parameter comparisons

To compare the performance of different variations of the LaDDR procedure, we generated DDPs for the Geuvadis dataset using each method and parameter variation (supplemental methods), for only protein-coding genes on chr7 for efficiency. We then mapped *cis*-QTLs for each of these phenotype sets using tensorQTL and used the number of conditionally independent *cis*-QTLs as a performance metric.

To compare LaDDR and Pantry runtime and memory usage, we ran both pipelines on the same 100 Geuvadis samples as were used for the read-length and -depth simulations. We used the snakemake benchmark feature to record time and memory metrics for each job and then summed the cpu_time values and took the maximum max_rss value per pipeline.

Functional enrichment analysis of fine-mapped xQTLs

For all RNA phenotypes with xQTLs from hybrid cross-modality mapping in each of 49 GTEx tissues, we applied SuSiE[30] fine-mapping via tensorQTL in cis_susie mode to the same input data. In each tissue, the RNA phenotype with the strongest xQTL per gene was fine-mapped. We measured functional enrichment on variants in the 95% credible sets for xQTLs of each modality, combined across all tissues. We downloaded variant annotations from the GTEx Portal (https://storage.googleapis.com/adult-gtex/references/v8/reference-tables/WGS_Feature_overlap_collapsed_VEP_short_4torus.MAF01.txt.gz). For the credible set variants of each modality, the fraction of variants in the credible sets annotated with each category was computed, weighted by posterior inclusion probability (PIP), and divided by the fraction of background variants annotated. The background set included all variants in the ± 1 Mb *cis*-windows of phenotyped genes. Enrichment values for which the weighted count was <0.1 were excluded due to low information.

Colocalization between xQTLs and FinnGen GWAS

We performed credible-set-based colocalization between the above fine-mapped xQTLs and FinnGen R12[31] fine-mapped associations for 1,302 traits by using SuSiE posterior inclusion probabilities. For each pair of overlapping xQTL and FinnGen credible sets, variants were matched by chromosome, position, reference allele, and alternative allele. We computed an eCAVIAR-style colocalization posterior probability, CLPP, as the sum of the products of xQTL and FinnGen posterior probabilities across shared variants and used CLPP > 0.01 as the colocalization threshold.

Colocalization for held-out modality analysis

We performed fine-mapping as described above on the Geuvadis hybrid (KDP + rDDP) cross-modality-mapped xQTLs, except that every RNA phenotype with an xQTL was fine-mapped to allow for colocalization tests across modalities. We computed CLPP and causal posterior agreement (CLPA) between each KDP xQTL and each rDDP xQTL of the same gene and determined significant colocalizations as those with CLPP > 0.01 or CLPA > 0.5. We tested colocalization in the same way between each modality of KDP

xQTLs and rDDP xQTLs resulting from holding out that KDP modality and used the increase in colocalizations as a conservative estimate of high-confidence recaptured xQTLs.

Intron retention quantification using MAJIQ

Retained introns were quantified in the Geuvadis RNA-seq samples via MAJIQ[32] v.3 with GENCODE v.47 gene annotations and –min-experiments of 0.01. For each retained intron, we tested correlations against same-gene rDDPs and KDPs across shared samples. Spearman correlation was computed for each retained-intron/phenotype pair, and Spearman p values were adjusted by Benjamini-Hochberg FDR across all tested pairs for each phenotype set. High-correlation retained-intron matches were defined as pairs with an absolute Spearman correlation of at least 0.5 and a Spearman FDR of at most 0.05.

Results

Overview of the method

The full LaDDR phenotyping procedure has four main steps (Figure 1A; methods).

(1) Get RNA-seq coverage. RNA-seq reads for a set of samples from one or more tissues are mapped to the genome and converted to base-level coverage. The products of these standard processing steps are often precomputed and freely available for download, for example through recount3,[23] even for datasets whose raw data are access controlled.
(2) Partition genes into bins. Each gene region is partitioned into bins used to summarize RNA-seq coverage. These bins are defined to segment the genome based on variation in RNA-seq coverage, grouping neighboring bases with similar coverage patterns across the reference data. We discuss different binning approaches in a subsequent section.
(3) Bin and normalize coverage. RNA-seq coverage is averaged per bin per sample and then normalized.
(4) Transform to DDPs. PCA is applied to those values per gene, and the sample projections onto each PC are saved as DDPs. These DDPs are comparable to KDPs, such as the multimodal set of phenotypes produced by Pantry, in that a number of phenotypes are produced per gene (Figure 1B). This allows many of the same applications of KDPs, including genetic association methods such as xQTL mapping and xTWAS, which can operate on multiple features per gene (Figure 1C).

The PCA models can then be applied to processed RNA-seq coverage data of additional datasets, and the resulting DDPs quantified for each gene will correspond (i.e., represent the same dimensions of transcriptomic variation) across all datasets generated from the same set of models. The coverage bin definitions can also be reused to generate new models on new data.

Benchmarking strategy and optimization of LaDDR parameters

We implemented multiple strategies for segmenting gene regions into bins to summarize coverage data. These include fixed numbers or widths of equal-sized bins per coding and non-coding region of each gene, as well as data-driven methods that aim to divide regions with greater coverage variation into more bins (supplemental methods). We applied each of these binning methods by using a variety of density parameters and mapped and quantified independent *cis*-xQTLs on the resulting DDPs to compare their effect (see supplemental methods). In general, the data-driven binning methods based on coverage variance performed best in terms of producing the most xQTLs and making efficient use of fewer bins (Figure S1A).

In the top-performing approach, which has been set as the default and used for the results presented in this study, the RNA-seq coverage values for a gene are $\log_2$ transformed, the difference in coverage between consecutive bases is computed, and the variance of these differences is computed at each base. Bin boundaries are then determined such that the cumulative sum of variance differences within each bin reaches a predefined threshold. This produces a higher density of smaller bins in gene regions and genes with more transcriptional variation than other regions and genes.

By default, PCA is applied to the binned normalized coverage data per gene, and the sample projections onto each PC are saved as DDPs. Functional PCA (FPCA) is also implemented as an alternative to PCA (supplemental methods). We tried different forms of FPCA, as well as PCA with different maximum numbers of PCs. The PCA runs produced more xQTLs than the FPCA runs, and as the maximum PC threshold increased, the number of resulting xQTLs increased and then decreased (Figure S1B). We therefore chose PCA with up to 16 PCs per gene as the default.

LaDDR phenotypes capture familiar and novel variation

We trained LaDDR gene models using 28,637 samples from 54 human tissues from the GTEx Project[33] and 33 human cancer types from TCGA.[34] These datasets provide RNA-seq coverage patterns across a diverse set of healthy and dysregulated tissue contexts. We applied the models to generate DDPs for each of 49 GTEx tissue datasets, producing an average of 417,593 (SD: 9,274) phenotypes per tissue across an average of 40,147 (SD: 1,351) protein-coding and lncRNA genes. We also trained and applied models on 445 human LCL samples from the Geuvadis dataset.[2]

We examined the DDPs in terms of their correlation with knowledge-driven phenotypes (KDPs) generated by Pantry. While some DDPs correlate primarily with a single KDP, such as total expression, more commonly they show varying degrees of correlation with multiple KDPs for the same gene (Figure 2A). We also assessed *cis*-heritability and found that it is, on average, highest for DDP1, the first

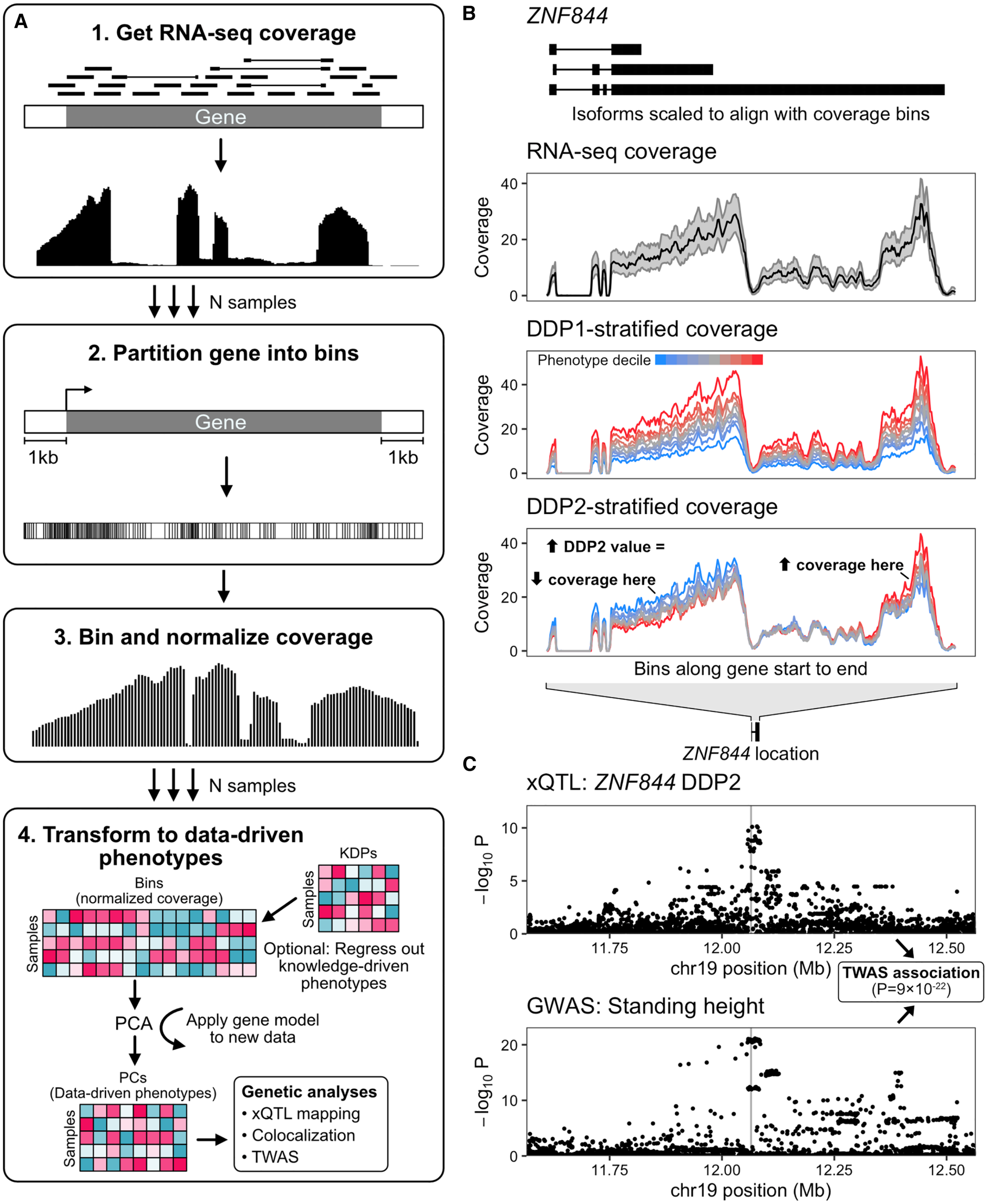

Figure 1. Overview of LaDDR phenotyping
(A) Diagram of the LaDDR procedure.
(B) Example of DDPs for one gene, *ZNF844*. Top: the three annotated isoforms of *ZNF844* are shown, and, as in the two images below, the *x* axis represents the coverage bin index along the gene and is not proportional to genomic position, as the actual bin widths vary. Isoforms are shown here for reference and are not used by LaDDR. Middle: RNA-seq coverage distribution (median and interquartile range) at each bin across all tibial nerve samples. Bottom: DDPs were generated using LaDDR. Tibial nerve samples were grouped into 10 equal-sized deciles based on their values for two resulting *ZNF884* phenotypes, DDP1 and DDP2, and the median coverage for each bin is shown for each decile. Coverage in all three images was scaled per sample to adjust for sequencing depth.
(C) TWAS association for DDP2 in (B). Top: nominal *p* values for each variant in the ±500 kb *cis*-window from xQTL mapping of *ZNF844* DDP2 in tibial nerve are shown. The location of *ZNF844* is shown above for reference, and its transcription start site is indicated in the plots with a vertical line. Bottom: GWAS *p* values for standing height (UK Biobank) in the same window.

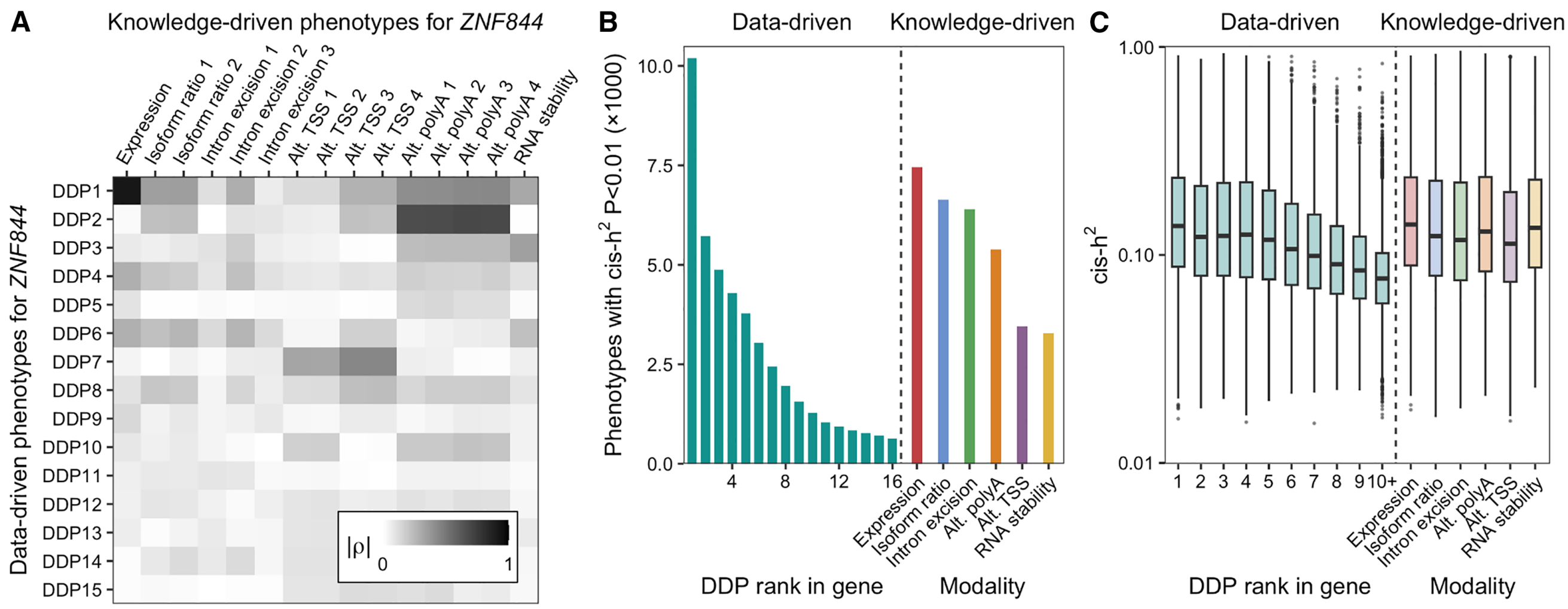

Figure 2. LaDDR phenotypes are similarly heritable to knowledge-driven phenotypes
(A) Heatmap of absolute value of Spearman correlations between each DDP (rows) and each KDP (columns) for one gene, *ZNF844*, across the Geuvadis dataset.
(B) Number of DDPs per DDP rank and KDPs per modality in the Geuvadis dataset that were used for xTWAS mapping. Only phenotypes with heritability $p < 0.01$ are used for xTWAS.
(C) Boxplots show *cis*-heritability of those heritable DDPs and KDPs.

PC for each gene, and decreases with increasing PC rank (Figure 2B). This pattern has a practical implication, as it supports reducing dimensionality by retaining only the leading DDPs. The *cis*-heritability of the earlier (lower-numbered) DDPs is comparable in distribution to the KDPs, while later (higher-numbered) DDPs have lower *cis*-heritability (Figure 2C).

LaDDR phenotypes encode multimodal genetic regulatory variation

We mapped *cis*-xQTLs (hereafter referred to as xQTLs) for the DDPs in each GTEx tissue using tensorQTL[27] in grouped phenotype mode with a 5% FDR significance threshold (see methods). We found 3,755–64,106 conditionally independent xQTLs per tissue for 3,419–29,007 genes (Table S1). We compared these results with those from Pantry, in which KDPs from six modalities were jointly mapped via cross-modality mapping, finding that, on average, 95% more independent *cis*-QTLs were identified for DDPs than for KDPs per tissue (Figures 3A and 3B).

DDPs corresponding to lower PCs of each gene PCA model tend to map more xQTLs than those corresponding to higher PCs, with DDP1 producing 7.4 times as many xQTLs as DDP8 and 18.6 times as many xQTLs as DDP16 (Figure 3C).

We assessed replicability of DDP xQTLs across independent datasets: GTEx LCL and Geuvadis LCL samples. For each GTEx LCL DDP xQTL lead variant, we examined the association for the same DDP-variant pair in Geuvadis, observing that 89% of the 15,871 pairs had the same effect direction (slope sign), with slopes correlating at Pearson $r = 0.67$ (Figure S2). Of the 60% of pairs also significant in Geuvadis, 99.7% had the same effect direction, with slopes correlating at Pearson $r = 0.94$.

We applied DDPs as transcriptomic models for xTWAS. Similar to the multimodal approach previously reported using Pantry phenotypes,[17] we used FUSION to fit a transcriptomic prediction model to each DDP in each tissue and then applied it to GWAS data for a collection of 114 complex traits.[7] Compared to six-modality xTWAS at the same p value threshold of 5×10^{-8} and using the same Geuvadis dataset for transcriptomic models, DDPs resulted in nearly the same number of total associations (24,906 versus 24,575 for six-modality TWAS), 35% more unique gene-trait pairs with associations (Figure 3D), and 38% more unique gene-trait pairs with strong evidence of colocalization at the level of shared causal variants (Figure 3E). Increases in unique gene-trait pairs were similar when using a threshold of 0.05 for Bonferroni-adjusted or Benjamini-Hochberg-adjusted p values for both sets of results instead of the 5×10^{-8} nominal threshold (33% increase in both cases; Figure S3). Applying xTWAS to DDPs for 49 GTEx tissues and the same 114 traits resulted in a median of 26,508 significant TWAS associations per GTEx tissue, including a total of 64,297 unique gene-trait pairs, 28,138 of which include strongly colocalizing associations.

LaDDR phenotypes can complement knowledge-driven phenotypes for genetic discovery

We developed a variant of the LaDDR phenotyping method that produces rDDPs, designed to complement KDPs and serve as a "catch-all" modality alongside them. This is accomplished by regressing out all KDPs from the normalized coverage features prior to PCA on a

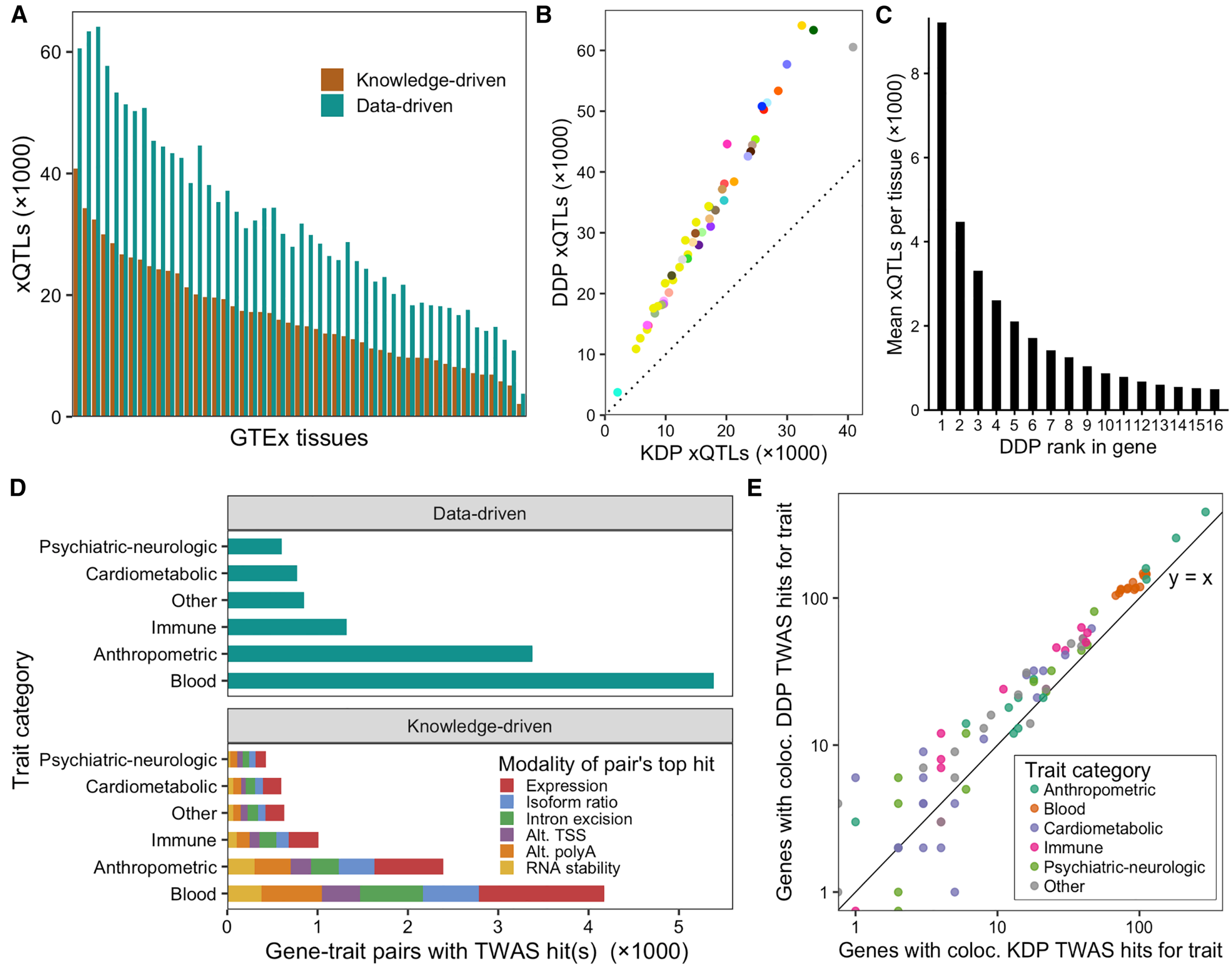

Figure 3. LaDDR increases *cis*-xQTL and xTWAS discovery
(A) xQTLs for DDPs and for KDPs in each GTEx tissue.
(B) The same values shown as a scatterplot, using the tissue colors provided by GTEx.
(C) Number of xQTLs, averaged over tissues, mapped for each within-gene DDP rank.
(D) Unique gene-trait pairs with xTWAS hits per trait category using DDPs (top) and KDPs (bottom).
(E) Number of unique gene-trait pairs with at least one colocalizing TWAS hit using DDPs (*y* axis) versus KDPs (*x* axis) for the transcriptomic models. Points are shown for the 86 tested traits with at least one colocalizing hit, and points with zero colocalizing hits for one of the axes are shown along an axis line to include them on the log-scaled axes.

per-gene basis (see methods). We produced rDDPs for Geuvadis using Pantry's six default modalities of KDPs and examined the maximum Pearson r^2 between each rDDP and a KDP of the same gene. For PC1, residualization reduced these values from a mean of 0.40 (SD: 0.27) to mean of 0.068 (SD: 0.093) (Figure 4A). For all PCs, residualization reduced the max r^2 values from a mean of 0.072 (SD: 0.14) to one of 0.030 (SD: 0.055).

To compare the ability of different sets of RNA phenotypes to represent *cis*-regulatory variation, we mapped *cis*-xQTLs that are conditionally independent across all phenotypes per gene using Pantry's cross-modality mapping procedure. We applied this to KDPs, DDPs, and a hybrid set of phenotypes (KDPs + rDDPs) for the Geuvadis dataset. Because we performed stepwise regression-based cross-modality mapping of this hybrid set, all reported KDP and rDDP xQTLs for the same gene in this analysis are conditionally independent from each other. Mapping only KDPs resulted in 22,470 total xQTLs, while mapping DDPs resulted in 30,158 xQTLs. Adding rDDPs to the set of KDPs as input to cross-modality mapping resulted in a net increase of 9,408 xQTLs (42%) for a total of 31,878, 16,544 of which were called for KDPs and 15,334 of which were called for rDDPs (Figure 4B). In terms of unique genes represented by the xQTLs, the addition of the rDDPs increased the number of xGenes from 12,872 to 16,950. Repeating the KDP, DDP, and hybrid xQTL mapping procedures for each GTEx tissue produced consistent results in terms of the relative number of independent xQTLs (Figure 4C; Table S1). All KDP xQTLs hereafter refer to those from the hybrid (KDP + rDDP) xQTL mapping.

To assess the ability of the rDDPs to capture modalities of transcriptomic variation that are not explicitly measured, we repeated the residual phenotyping procedure for the Geuvadis data, each time withholding one KDP modality and residualizing the normalized coverage

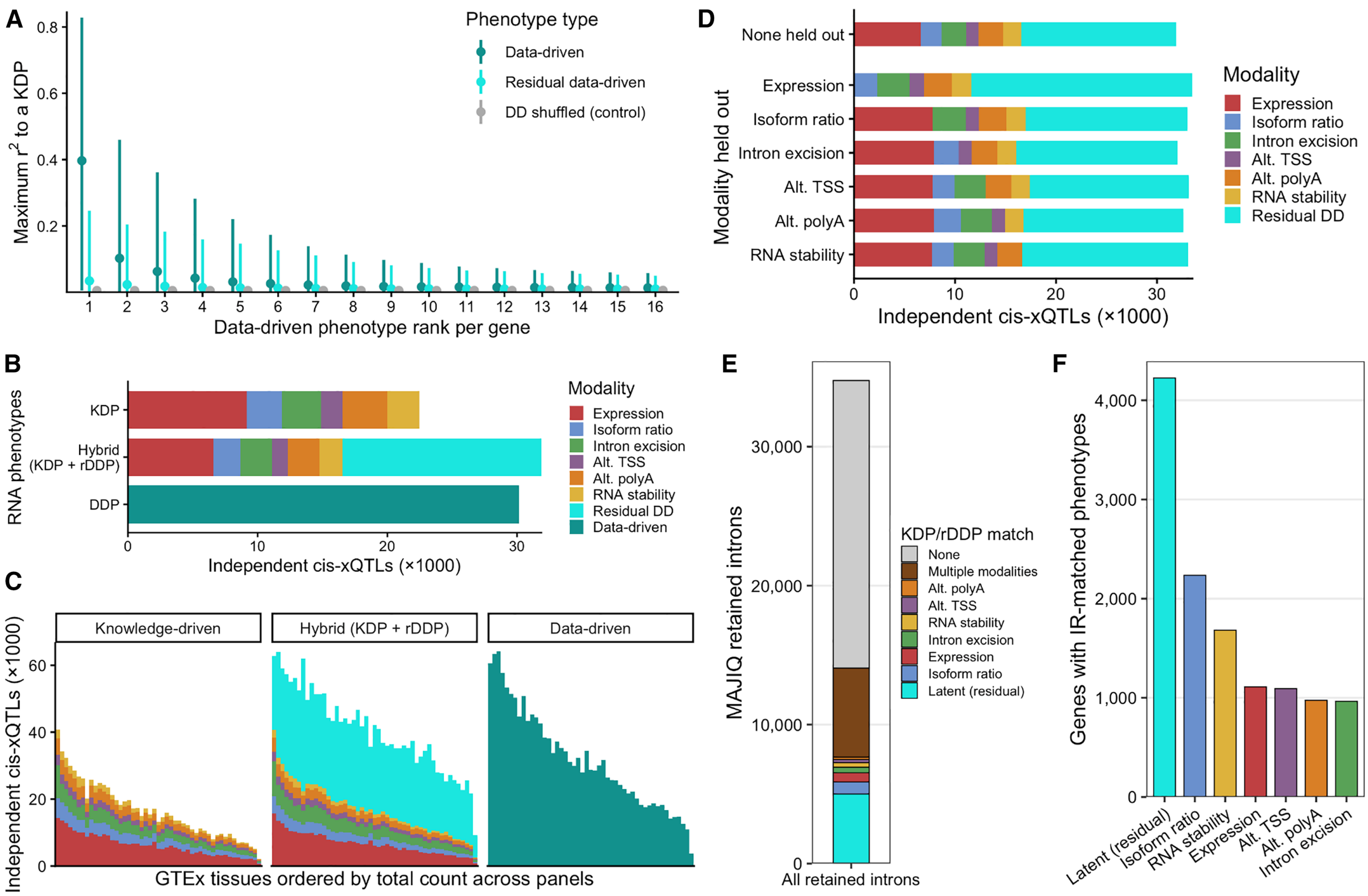

Figure 4. Residual data-driven RNA phenotypes complement knowledge-driven phenotypes

(A) Correlations between data-driven (full and residual) and knowledge-driven phenotypes for Geuvadis. As a control, values for each DDP were shuffled, and maximum r^2 with KDPs were recomputed. Points show medians and lines show 5% to 95% quantile ranges.
(B) Independent xQTLs produced by cross-modality mapping on six modalities of KDPs, cross-modality mapping on six modalities of KDPs plus rDDPs, and *cis*-QTL mapping on only DDPs for Geuvadis.
(C) Independent xQTLs from the same three forms of cross-modality mapping applied to each GTEx tissue.
(D) xQTLs from hybrid (KDP + rDDP) cross-modality mapping using all six KDP modalities and xQTLs resulting from hybrid cross-modality mapping where each modality of KDPs was individually held out from rDDP generation and xQTL mapping.
(E) Total number of retained intron phenotypes quantified by MAJIQ. Subsets of phenotypes with high Spearman correlation (|rho| > 0.5 and Spearman FDR ≤ 0.05) to phenotypes of a KDP modality, rDDP, or multiple modalities (including rDDP) are colored.
(F) Number of RNA phenotypes of each modality with high correlation to a MAJIQ retained intron phenotype.

data on the other five. We then repeated hybrid cross-modality mapping, each time using phenotypes for only five KDP modalities plus the rDDPs residualized on those five modalities. In every case, holding out a single modality increased the number of rDDPs in compensation, yielding a similar total number of independent xQTLs (Figure 4D).

We examined the additional rDDP xQTLs obtained by holding out each KDP modality. Since the exact correspondence of xQTLs across different xQTL mapping results is not straightforward, we examined only rDDP xQTLs for genes that lacked rDDP xQTLs in the non-held-out hybrid mapping. As expected, the positions of these xQTLs relative to their xGene somewhat reflect the modality that was held out (Figure S4). As a conservative but more direct test of LaDDR's ability to capture xQTLs for each modality, we performed fine-mapping and colocalization between each modality's xQTLs and rDDP xQTLs before and after withholding the modality. In total, 4,970 xQTLs from the held-out modalities colocalized to post-withholding rDDP xQTLs, compared to 635 xQTLs colocalized to pre-withholding xQTLs, an increase of 4,335. Per modality, this increase ranged from 47 for alternative TSS to 3,160 for expression.

We also assessed LaDDR's ability to capture a particular form of variation, intron retention (IR), which can be missed by splicing quantification methods when not explicitly annotated. We used MAJIQ[32] v.3 to quantify IR in Geuvadis samples and matched them to rDDPs and KDPs based on Spearman correlation (|rho| > 0.5 and Spearman FDR ≤ 0.05). Of the 34,752 retained introns detected and quantified, 10,502 matched with one or more rDDPs (Figure 4E). There were 4,225 genes with IR-matched rDDPs, while the greatest number of such genes for a KDP modality was 2,236 genes with IR-matched isoform ratio phenotypes (Figure 4F). Of the IR-rDDP matches, 1,205 had xQTLs, and 623 had xTWAS hits.

We then examined the application of rDDPs to xTWAS. Unlike cross-modality-mapped xQTLs,

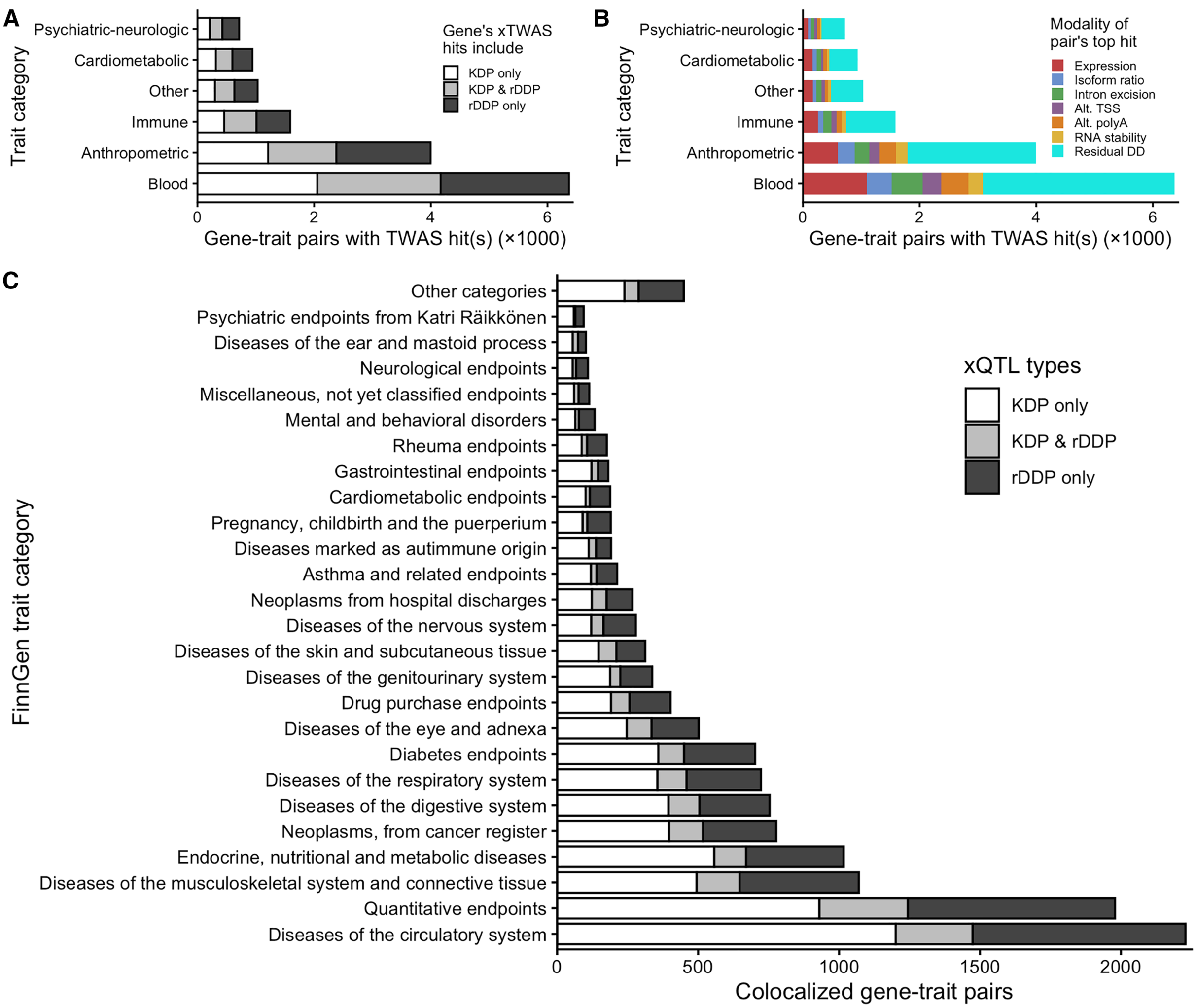

Figure 5. Residual data-driven RNA phenotypes expand gene-trait association discovery
(A) Number of unique gene-trait pairs with hybrid (KDP + rDDP) xTWAS hits per trait category. Pairs are colored by whether the associated phenotypes per gene-trait pair included one or more KDPs, one or more rDDPs, or both.
(B) The same gene-trait pairs as in (E) but grouped and colored by the modality of the hit with the lowest p value.
(C) For each FinnGen-assigned trait category, the number of unique colocalized gene-trait pairs is shown. Pairs are colored by whether their significant colocalizations involved KDP xQTLs only, rDDP xQTLs only, or both, and these include colocalizations in any GTEx tissue. All trait categories besides the 25 with the most colocalized gene-trait pairs are grouped into "other categories."

conditional independence across phenotypes and modalities is not enforced by the TWAS method used in this study, so we ran TWAS on the rDDPs for Geuvadis and simply combined the results with those from KDPs. This resulted in a 59% increase in unique gene-trait association pairs (Figure 5A), and rDDPs showed the strongest associations for 7,774 (53%) of the pairs (Figure 5B). Similarly, across GTEx tissues, adding rDDP TWAS hits increased the average number of unique gene-trait association pairs by 58% (Table S2), and rDDPs had the strongest associations for 6,913 pairs (51%) on average. Looking at the subset of xTWAS hits with strong evidence of colocalization at the shared causal variant level, adding rDDP hits increased gene-trait pairs by 63% per tissue, and rDDPs had the strongest associations for 2,340 pairs (49%) on average.

Of particular interest are gene-trait pairs that have xTWAS hits for both DDPs and rDDPs but no hits for KDPs in any tissue. Four such gene-trait pairs are highlighted here (Figure S5).

- The "morning/evening person (chronotype)" trait had data-driven xTWAS hits for *CCNH* (cyclin H) in adrenal gland tissue (lead variant chr5:87375153 [GRCh38] for both xQTL and GWAS). *CCNH* was previously mapped to a GWAS locus for chronotype,[35] and cyclin-CDK complexes, which control cell cycle progression, have been shown to interact with circadian rhythm machinery.[36]
- The "triglycerides" trait had data-driven xTWAS hits for *RFLNA* (refilin A) in left ventricle tissue (lead variants chr12:123929551 for xQTL and

chr12:123980289 for GWAS). *RFLNA* was previously mapped to a GWAS locus for triglyceride levels.[37]

- The "fasting glucose" trait had a data-driven xTWAS hit for *DGKB* (diacylglycerol kinase beta) in pancreas (lead variants chr7:14887046 for xQTL and chr7:15025378 for GWAS). *DGKB* was previously mapped to a GWAS locus for type 2 diabetes mellitus[38] and is involved in glucose homeostasis.[39]
- Finally, the "sleeplessness/insomnia" trait had a data-driven xTWAS hit for *LINC03051* (long intergenic non-protein coding RNA 3051) in the anterior cingulate cortex (BA24) (lead variants chr3:117908698 for xQTL and chr3:117918850 for GWAS). *LINC03051* was previously mapped to a GWAS locus for insomnia.[40]

While experiments would be needed to determine causal relationships, these examples bolster prior GWAS discoveries of candidate mediators and demonstrate how gene-trait genetic associations missed by knowledge-driven phenotyping can be captured by LaDDR phenotyping.

To validate the overall trends observed when applying xTWAS to LaDDR phenotypes, we performed colocalization using different methodologies and GWAS data. We computed colocalization posterior probabilities between fine-mapped KDP or rDDP xQTLs and fine-mapped GWAS variants for 1,302 traits from FinnGen[31] (Table S3). Of the 13,489 gene-trait pairs with significant colocalizations, 35% included only rDDP xQTLs, 14% included rDDP and KDP xQTLs, and 51% included only KDP xQTLs (Figures 5C and S6).

Functional characteristics of phenotypes and their genetic associations

We aggregated the positions of KDP, rDDP, and DDP xQTLs relative to their xGenes and found that the distributions of both rDDP and DDP xQTLs exhibited a mixture of the major features of the KDP xQTLs, namely concentration within the gene body with peaks at the gene start and end (Figure 6A). DDP xQTLs, which were mapped on their own, showed a stronger peak at the gene start, while rDDP xQTLs, which were residualized using KDPs and included in hybrid cross-modality mapping alongside KDPs, had a lower peak at the gene start than at the gene end.

We then measured enrichment of various functional annotations in the fine-mapped xQTLs of each modality. For most functional categories, rDDP xQTLs were enriched but less so than any knowledge-driven modality (Figure 6B). However, when filtering xQTLs to those for RNA phenotypes that also had colocalizing xTWAS hits in the same tissue, functional enrichment in rDDP xQTLs was similar to that of knowledge-driven modalities (Figure 6C).

We also compared the relative rate of colocalization with GWAS loci per xQTL for rDDPs and KDPs. Expression had the lowest ratio of colocalizing TWAS hits to xQTLs (median of 0.17 across tissues), and rDDPs had the second-lowest ratio (median of 0.27; Figure 6D–6F). Expression and rDDPs also had the lowest ratios for unique genes with colocalizing TWAS hits per unique xQTL gene and for colocalizing TWAS hits per xQTL when filtered to the top hit per gene-trait pair and top xQTL per gene (Figure S7).

To check for indications that DDPs could be more susceptible to false positives due to misassignment of RNA-seq coverage to nearby genes, we measured the rate at which genes in close proximity were associated with the same trait, noting that such cases may also have other causes, such as pleiotropy and genuine independent mediators. Defining nearby as having any overlap according to the gene's transcription start and end positions in the gene annotations, 35.5% of DDP TWAS gene-trait pairs for protein-coding genes had a nearby gene associated with the same trait, compared to 31.4% of KDP TWAS pairs (Figure S8). For gene-trait pairs involving lncRNAs, the percentages were higher, at 60.2% for DDP TWAS and 57.7% for KDP TWAS. The similarity of these percentages for DDPs versus KDPs suggests that misassigned RNA-seq coverage is not substantially higher in LaDDR phenotyping than in the more established RNA phenotyping methods. Stranded RNA-seq data can help resolve coverage assignment when nearby genes are on opposite strands, so LaDDR has an option to provide a pair of bigWig coverage files per sample containing plus/minus strand coverage. Then, for each gene, LaDDR will only load coverage on the gene's strand.

Despite lncRNAs having higher transcript complexity (i.e., splice variants per exon) than protein-coding mRNAs,[41] xQTL mapping across six KDP modalities produced an average of 0.16 independent xQTLs per gene per tissue for lncRNAs, which is only 29% of the rate for protein-coding genes. Multiple biological and methodological factors could be contributing to this difference, but gene annotation completeness could be one factor. In the annotations used in this study, lncRNAs have 28.9% of the number of exons per gene summed over isoforms compared to protein-coding genes. However, DDP xQTL mapping showed less of a difference, with 0.39 xQTLs per lncRNA per tissue, which was 44% of the rate for protein-coding genes.

Impact of input data quality

DDPs from LaDDR are robust across datasets and library types and are minimally impacted by missing annotations. We quantified this robustness by running LaDDR using different amounts of training data, simulated sparse reference annotations, and simulated variation in input RNA-seq read types and lengths.

Because some transcriptomic variation is tissue specific, we hypothesized that fitting LaDDR gene models using more samples from more tissues and other contexts would result in phenotypes that capture more regulatory

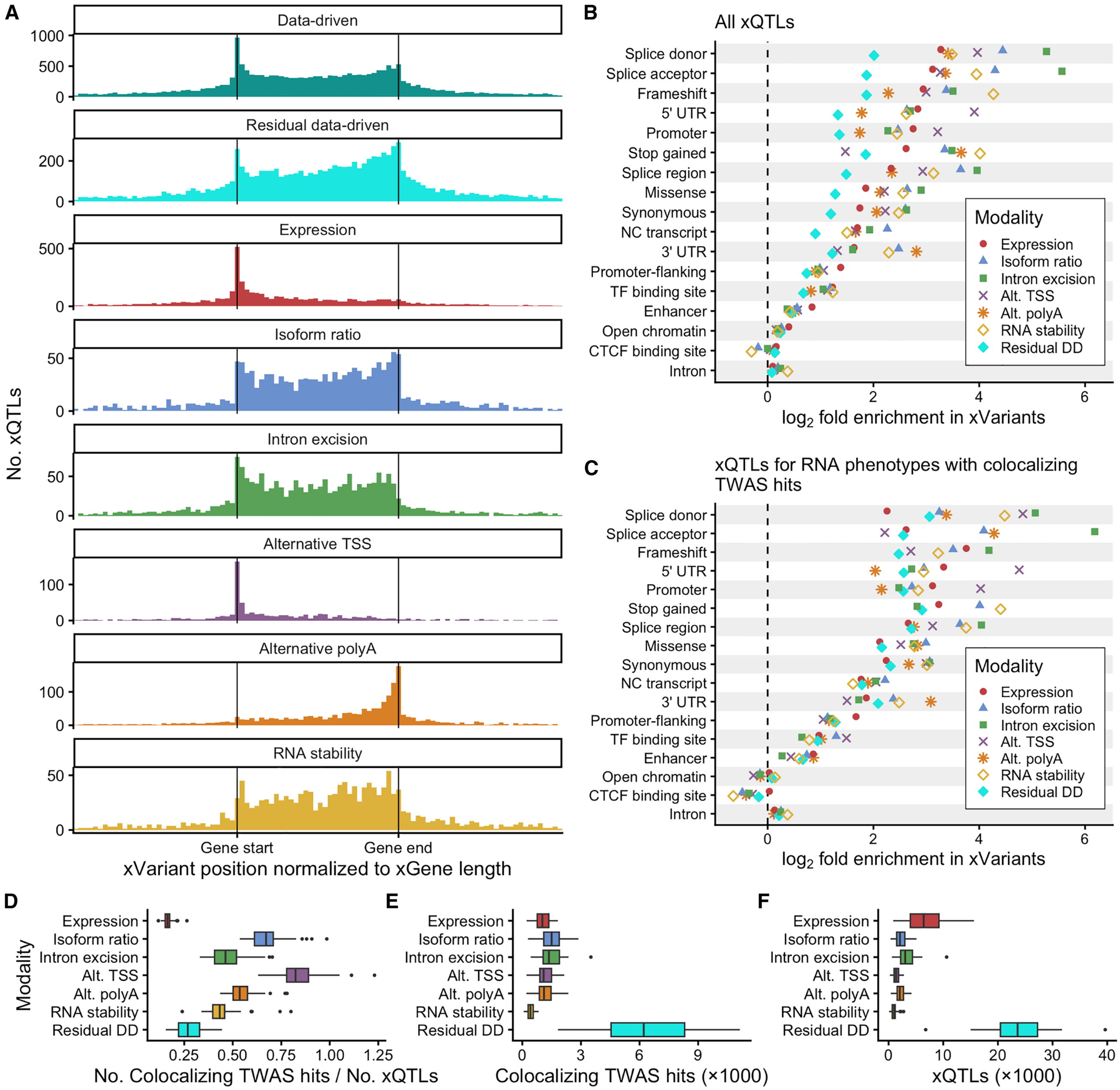

Figure 6. Functional characteristics of LaDDR phenotyping

(A) Histograms of DDP, rDDP, and KDP xQTL locations relative to their xGene. The first image shows xQTLs from DDP xQTL mapping, and the remaining seven images show xQTLs from hybrid xQTL mapping across all KDPs and rDDPs. The genomic coordinates of each xGene and that gene's xQTLs were linearly transformed such that the gene starts and ends are aligned on the *x* axis. xQTLs more than one gene length from the gene are not shown. Histograms are colored by modality.

(B) Enrichment of functional categories in the fine-mapped xQTLs from hybrid cross-modality mapping. For each modality of xQTLs, the $\log_2$ ratio of the prevalence of each annotation among fine-mapped xQTLs to the background prevalence is shown. Enrichment was computed for xQTLs aggregated across 49 GTEx tissues. Categories are sorted by expression modality enrichment value.

(C) Enrichment was computed the same way as in (B) but after filtering xQTLs to those for RNA phenotypes with at least one colocalizing TWAS hit in the same tissue.

(D) For each modality, the number of colocalizing xTWAS hits, divided by the number of xQTLs from hybrid cross-modality mapping, was computed for each GTEx tissue and shown as a boxplot.

(E) The numerators for the ratios plotted in (D) are shown as boxplots.

(F) The denominators for the ratios plotted in (D) are shown as boxplots.

variation. We fitted a set of models using five GTEx tissues from different tissue categories (adipose - subcutaneous, artery - aorta, brain - frontal cortex [BA9], esophagus - gastroesophageal junction, and skin - sun exposed [lower leg]), a separate set of models using all 54 GTEx tissues, and a third set of models using all 54 GTEx tissues and

33 cancer types from TCGA.[34] We compared them by using each set to generate DDPs for 49 GTEx tissues and map *cis*-xQTLs for each tissue for each set of models. Unexpectedly, using 54 rather than five tissues to fit models increased the number of independent xQTLs per tissue by only 4.2% on average (Figure S9A). Using 54 tissues plus 33 cancer types increased xQTLs by 1.7% on average compared to 54 tissues only (Figure S9B). In other words, xQTLs for a tissue can be found at nearly the same rate regardless of whether that particular tissue was used to fit the LaDDR models.

To measure the effect of gene annotation sparsity on the ability for DDPs to represent regulatory variation, we generated a series of increasingly sparse annotations by dropping non-canonical isoforms in random order until only the canonical isoform for each gene remained. We used each set of gene annotations to generate DDPs with LaDDR and KDPs with Pantry and then mapped xQTLs for the phenotypes. DDPs were less affected by gene annotation sparsity than were KDPs: independent xQTLs dropped 1.8% between 100% and 0% inclusion of non-canonical isoforms, compared with a 14% drop for KDPs (Figure S10).

To estimate the effect of RNA-seq technologies on LaDDR phenotyping quality, we selected 100 Geuvadis samples with 75 bp paired-end RNA-seq and altered the reads in three ways. These included truncating all reads to 50 bp, treating paired-end reads as single end, and subsampling reads to fractions of the original depths. When we used each of these variations as input for LaDDR and mapped *cis*-QTLs to the DDPs, we observed a relatively small drop (−2.7%) in discoveries from the 50 bp truncated reads and larger drops from the single-end (−7.2%) and lowered sequencing depth (−41% for 50% of reads and −66% for 25% of reads) simulations (Figure S11).

We measured CPU time and memory usage of LaDDR and Pantry on the same 100 Geuvadis samples, starting from FASTQ files and including STAR read mapping for both methods. LaDDR took 209 CPU hours to generate DDPs, including generating bins and fitting models. Pantry took 329 CPU hours to generate KDPs, though most of that was building index files for txrevise, which are computed once and can be reused for any number of samples and datasets. Excluding STAR read mapping, the maximum memory usage was 3.1 GB for LaDDR and 7.8 GB for Pantry.

Discussion

We introduced LaDDR, a method for data-driven RNA phenotyping that efficiently captures regulatory variation with minimal dependence on reference gene annotations. We showed that DDPs encode more regulatory variation than six major modalities of KDPs combined, both in terms of the number of independent *cis*-xQTLs and the number of gene-trait xTWAS associations discovered. We also described rDDPs that, when combined with a set of knowledge-driven phenotypes, increased the discovery of independent *cis*-xQTLs by 42% and the number of gene-trait pairs with xTWAS associations by 37%. While DDPs are generally less functionally interpretable than KDPs, DDPs trade off this limitation with the advantage of encoding more cumulative genetic regulatory variation, while rDDPs serve the purpose of being used in combination with KDPs to maintain interpretable genetic associations with KDPs and supplement them with additional discoveries.

Though LaDDR joins existing methods such as SCISSOR[18] and derfinder[20] in extracting features from base-level RNA-seq coverage data, it was built to enable direct application to population genetic analyses. Thus, rather than focusing on outliers or differential expression, it produces molecular phenotypes that can be genetically mapped in the same way as knowledge-driven molecular phenotypes. The strong replication of xQTLs in the GTEx/Geuvadis LCL analysis suggests that these DDPs represent true biological features rather than cohort-specific artifacts.

While some methods for latent embeddings of genomic data use deep learning,[42–44] the approach introduced here uses features that are linearly related to log-transformed expression coverage. One advantage of this approach is a straightforward characterization of phenotypes by examining the PCA loadings for the coverage bins across the gene, revealing which portions of the gene vary in proportion to that phenotype's values. Another advantage is that the PCs represent orthogonal dimensions of variation, providing a compact set of features that minimize redundancy in their resulting genetic associations and overfitting.

Two analyses together suggest why LaDDR discovers more gene regulatory associations than the panel of KDPs used in this study. The analysis in which KDPs were held out of rDDP residualization and xQTL mapping one modality at a time (Figure 4D) showed that DDPs from LaDDR can encode regulatory variation in KDPs, so long as they are not intentionally regressed out. The analysis comparing the number of LaDDR DDP xQTLs versus KDP xQTLs using subsampled isoform annotations (Figure S10) showed that LaDDR is minimally impacted by annotation sparsity. Assuming the currently known gene annotations are incomplete, these observations suggest that LaDDR DDPs encode regulatory variation that could be identified in KDPs using future annotations. However, the leveling off of KDP xQTLs in the subsampled isoform annotations analysis, far below the number of DDP xQTLs, suggests that DDPs capture regulatory variation beyond what the KDP modalities implemented by Pantry can capture, even with fully comprehensive annotations. These two interpretations suggest that DDPs can capture two forms of transcriptomic variation missing from a collection of KDPs: uncharacterized modalities of variation and specific instances of variation in known

mechanisms that were not represented in the reference gene annotations. Both of these benefits may decrease as mechanistic knowledge and transcriptome annotation increase. Still, the compactness and computational efficiency of DDPs may remain advantageous for some applications.

We also showed that, despite relying minimally on gene annotations and no modality-specific knowledge or methodology, the use of DDPs for xTWAS resulted in more discovered gene-trait associations than xTWAS with six transcriptomic modalities using Pantry. Another advantage of using PCA-derived DDPs for xTWAS is the reduction of redundant associations arising from correlated phenotypes for the same gene. Still, when maximal discovery of gene-trait associations and potentially implicated modalities is a priority, a hybrid set of phenotypes (KDPs + rDDPs) can be used.

We found that the LaDDR method presented here is more effective at quantifying regulatory variation in lncRNAs than the six KDP modalities we tested. Given the high level of genetic variation within lncRNAs and their high diversity of functions,[45] the ability to capture lncRNA regulatory variation is an important component of transcriptome-wide genetic analyses. Nevertheless, the inclusion of non-coding transcripts poses challenges for avoiding misassignment of RNA-seq reads to nearby or overlapping gene regions. In the current LaDDR implementation, we address this by excluding regions from one gene that overlap with annotated exonic regions of another gene for the purposes of RNA-seq coverage quantification. Refinements of this procedure, such as a probabilistic method of gene assignment, could further reduce potential misassignment of coverage variation while maximizing the inclusion of coverage data for each gene. Still, our analysis of nearby genes sharing DDP xTWAS associations showed only a slight increase in such cases compared to KDP xTWAS. Strand-specific coverage data, when available, can be used in LaDDR to improve the assignment of coverage to genes in problematic regions.

For most variant functional categories, we observed less enrichment in rDDP xQTLs than in KDP xQTLs (Figure 6B), though this difference was diminished after filtering to RNA phenotypes with colocalizing TWAS hits (Figure 6C). One hypothesis is that variant annotations correspond somewhat to known gene annotations, and so RNA phenotypes residualized to remove variation quantified using known annotations will associate more with variants that are unannotated. However, it could also indicate that rDDPs produce a higher fraction of xQTLs that are in some way spurious than those produced by KDPs. One potential source of spurious xQTLs is reference mapping bias, which is known to affect transcriptomic quantification methods[46] and could alter RNA-seq coverage in correlation with a variant. Further research would be needed to assess these hypotheses. A related observation was that while rDDPs exhibit a relatively low ratio of colocalizing TWAS hits to xQTLs, eQTLs have an even lower ratio. Given that rDDPs and expression produced more xQTLs than the other modalities, their power may result in detection of a higher fraction of weaker regulatory effects that are less likely to associate strongly with complex traits.

We found that LaDDR models built with 54 tissue datasets produced slightly more xQTLs than those built with 5 tissue datasets, and adding 33 cancer datasets provided a slight additional improvement. We speculate that models trained on multiple tissues do not capture much of the tissue-specific variation patterns, especially because only genes expressed in most of the full set of training samples are included, thereby omitting genes with highly tissue-specific expression. Future versions of LaDDR could explore whether fitting tissue-specific or other context-specific models results in an increase in tissue specific, and thus an increase in total, genetic associations, and whether that benefit outweighs potential drawbacks such as lower sample size per model set, greater computational burden, and the loss of correspondence of phenotype representations across tissues.

Since our model training comparison showed that LaDDR can effectively generate phenotypes for a tissue even without being trained on data from that tissue, there are multiple usage options. LaDDR can be run using downloaded existing gene bin definitions, or they can be computed from a new set of RNA-seq coverage files (which is required if using a different species or genome build than what is available). If existing bins are used, LaDDR models can also be downloaded and reused or generated from new data. If there is a desire for latent phenotype IDs to correspond with existing data, the same models should be reused. Otherwise, we recommend computing new models to ensure that transcriptional variation in the new data is maximally captured. The model-fitting stage is a small fraction of the LaDDR runtime, which is dominated by processing coverage data used for both model fitting and phenotype generation, and requires no extra setup. In either case, we recommend using existing gene bin definitions.

LaDDR greatly expands the information extracted from RNA-seq data that can be applied to genetic analyses. We provide DDPs, rDDPs, and the resulting xQTLs and TWAS weights and associations in the Pantry Portal (https://pantry.pejlab.org/) alongside their knowledge-driven counterparts. We also provide the LaDDR code as a Python package (https://github.com/PejLab/LaDDR) and have integrated it into the Pantry framework (https://github.com/PejLab/Pantry) to facilitate the joint use of knowledge-driven and data-driven RNA phenotypes to enhance genetic discovery.

Data and code availability

The accession number for the LaDDR phenotypes, models, xQTLs, and xTWAS results for GTEx and Geuvadis data reported in this paper is Zenodo: 19924423 (https://doi.org/10.5281/zenodo.

19924423). Transcriptomic model weights for running xTWAS are available for all DDPs, rDDPs, and KDPs for GTEx tissues and Geuvadis at https://pantry.pejlab.org/download. GWAS summary statistics used for xTWAS were downloaded from Zenodo: 3629742 (https://doi.org/10.5281/zenodo.3629742).[28] LaDDR is implemented as a Python package available at https://github.com/PejLab/LaDDR.[47] Code used to run LaDDR and other analyses for this study is available at https://github.com/daniel-munro/laddr-cluster. Code for final data processing and for generating figures, tables, and statistics is available at https://github.com/daniel-munro/laddr-paper.

Acknowledgments

We want to acknowledge the participants and investigators of the FinnGen study. This work was funded by the National Institute on Drug Abuse (P30DA060810 to A.A.P. and U01DA060443 to P.M.).

Declaration of interests

The authors declare no competing interests.

Supplemental information

Supplemental information can be found online at https://doi.org/10.1016/j.ajhg.2026.06.005.

Received: March 17, 2026
Accepted: June 7, 2026
Published: July 2, 2026

References

1. Montgomery, S.B., and Dermitzakis, E.T. (2011). From expression QTLs to personalized transcriptomics. Nat. Rev. Genet. *12*, 277–282. https://doi.org/10.1038/nrg2969.
2. Lappalainen, T., Sammeth, M., Friedländer, M.R., t Hoen, P.A.C., Monlong, J., Rivas, M.A., Gonzàlez-Porta, M., Kurbatova, N., Griebel, T., Ferreira, P.G., et al. (2013). Transcriptome and genome sequencing uncovers functional variation in humans. Nature *501*, 506–511. https://doi.org/10.1038/nature12531.
3. Battle, A., Mostafavi, S., Zhu, X., Potash, J.B., Weissman, M.M., McCormick, C., Haudenschild, C.D., Beckman, K.B., Shi, J., Mei, R., et al. (2014). Characterizing the genetic basis of transcriptome diversity through RNA-sequencing of 922 individuals. Genome Res. *24*, 14–24. https://doi.org/10.1101/gr.155192.113.
4. Jones, E.F., Haldar, A., Oza, V.H., and Lasseigne, B.N. (2024). Quantifying transcriptome diversity: a review. Brief. Funct. Genomics *23*, 83–94. https://doi.org/10.1093/bfgp/elad019.
5. Gamazon, E.R., Wheeler, H.E., Shah, K.P., Mozaffari, S.V., Aquino-Michaels, K., Carroll, R.J., Eyler, A.E., Denny, J.C., GTEx Consortium, and Nicolae, D.L., et al. (2015). A gene-based association method for mapping traits using reference transcriptome data. Nat. Genet. *47*, 1091–1098. https://doi.org/10.1038/ng.3367.
6. Gusev, A., Ko, A., Shi, H., Bhatia, G., Chung, W., Penninx, B.W.J.H., Jansen, R., de Geus, E.J.C., Boomsma, D.I., Wright, F.A., et al. (2016). Integrative approaches for large-scale transcriptome-wide association studies. Nat. Genet. *48*, 245–252. https://doi.org/10.1038/ng.3506.
7. Barbeira, A.N., Bonazzola, R., Gamazon, E.R., Liang, Y., Park, Y., Kim-Hellmuth, S., Wang, G., Jiang, Z., Zhou, D., Hormozdiari, F., et al. (2021). Exploiting the GTEx resources to decipher the mechanisms at GWAS loci. Genome Biol. *22*, 49. https://doi.org/10.1186/s13059-020-02252-4.
8. Zhao, S., Crouse, W., Qian, S., Luo, K., Stephens, M., and He, X. (2024). Adjusting for genetic confounders in transcriptome-wide association studies improves discovery of risk genes of complex traits. Nat. Genet. *56*, 336–347. https://doi.org/10.1038/s41588-023-01648-9.
9. Liu, D., Bae, Y.E., Zhu, J., Zhang, Z., Sun, Y., Deng, Y., Wu, C., and Wu, L. (2023). Splicing transcriptome-wide association study to identify splicing events for pancreatic cancer risk. Carcinogenesis *44*, 741–747. bgad069. https://doi.org/10.1093/carcin/bgad069.
10. Cui, Y., Arnold, F.J., Peng, F., Wang, D., Li, J.S., Michels, S., Wagner, E.J., La Spada, A.R., and Li, W. (2023). Alternative polyadenylation transcriptome-wide association study identifies APA-linked susceptibility genes in brain disorders. Nat. Commun. *14*, 583. https://doi.org/10.1038/s41467-023-36311-8.
11. Bhattacharya, A., Vo, D.D., Jops, C., Kim, M., Wen, C., Hervoso, J.L., Pasaniuc, B., and Gandal, M.J. (2023). Isoform-level transcriptome-wide association uncovers genetic risk mechanisms for neuropsychiatric disorders in the human brain. Nat. Genet. *55*, 2117–2128. https://doi.org/10.1038/s41588-023-01560-2.
12. Gao, G., McClellan, J., Barbeira, A.N., Fiorica, P.N., Li, J.L., Mu, Z., Olopade, O.I., Huo, D., and Im, H.K. (2024). A multi-tissue, splicing-based joint transcriptome-wide association study identifies susceptibility genes for breast cancer. Am. J. Hum. Genet. *111*, 1100–1113. https://doi.org/10.1016/j.ajhg.2024.04.010.
13. Zhang, D., Guelfi, S., Garcia-Ruiz, S., Costa, B., Reynolds, R.H., D'Sa, K., Liu, W., Courtin, T., Peterson, A., Jaffe, A.E., et al. (2020). Incomplete annotation has a disproportionate impact on our understanding of Mendelian and complex neurogenetic disorders. Sci. Adv. *6*, eaay8299. https://doi.org/10.1126/sciadv.aay8299.
14. Glinos, D.A., Garborcauskas, G., Hoffman, P., Ehsan, N., Jiang, L., Gokden, A., Dai, X., Aguet, F., Brown, K.L., Garimella, K., et al. (2022). Transcriptome variation in human tissues revealed by long-read sequencing. Nature *608*, 353–359. https://doi.org/10.1038/s41586-022-05035-y.
15. Fair, B., Buen Abad Najar, C.F., Zhao, J., Lozano, S., Reilly, A., Mossian, G., Staley, J.P., Wang, J., and Li, Y.I. (2024). Global impact of unproductive splicing on human gene expression. Nat. Genet. *56*, 1851–1861. https://doi.org/10.1038/s41588-024-01872-x.
16. Kerimov, N., Hayhurst, J.D., Peikova, K., Manning, J.R., Walter, P., Kolberg, L., Samoviča, M., Sakthivel, M.P., Kuzmin, I., Trevanion, S.J., et al. (2021). A compendium of uniformly processed human gene expression and splicing quantitative trait loci. Nat. Genet. *53*, 1290–1299. https://doi.org/10.1038/s41588-021-00924-w.
17. Munro, D., Ehsan, N., Esmaeili-Fard, S.M., Gusev, A., Palmer, A.A., and Mohammadi, P. (2024). Multimodal analysis of RNA sequencing data powers discovery of complex trait genetics. Nat. Commun. *15*, 10387. https://doi.org/10.1038/s41467-024-54840-8.

18. Choi, H.Y., Jo, H., Zhao, X., Hoadley, K.A., Newman, S., Holt, J., Hayward, M.C., Love, M.I., Marron, J.S., and Hayes, D.N. (2021). SCISSOR: a framework for identifying structural changes in RNA transcripts. Nat. Commun. *12*, 286. https://doi.org/10.1038/s41467-020-20593-3.
19. Jaffe, A.E., Shin, J., Collado-Torres, L., Leek, J.T., Tao, R., Li, C., Gao, Y., Jia, Y., Maher, B.J., Hyde, T.M., et al. (2015). Developmental regulation of human cortex transcription and its clinical relevance at single base resolution. Nat. Neurosci. *18*, 154–161. https://doi.org/10.1038/nn.3898.
20. Collado-Torres, L., Nellore, A., Frazee, A.C., Wilks, C., Love, M.I., Langmead, B., Irizarry, R.A., Leek, J.T., and Jaffe, A.E. (2017). Flexible expressed region analysis for RNA-seq with derfinder. Nucleic Acids Res. *45*, e9. https://doi.org/10.1093/nar/gkw852.
21. Ryan, D., Roberts, E., Eraslan, G., Grüning, B., D, W., Jamous, B.A., Betts, E., Ramirez, F., Fox, N., Abdennur, N., et al. (2024). deeptools/pyBigWig: 0.3.23. Version 0.3.23. Zenodo. https://doi.org/10.5281/zenodo.12158845.
22. Pedregosa, F., Varoquaux, G., Gramfort, A., Michel, V., Thirion, B., Grisel, O., Blondel, M., Prettenhofer, P., Weiss, R., Dubourg, V., et al. (2011). Scikit-learn: Machine Learning in Python. J. Mach. Learn. Res. *12*, 2825–2830.
23. Wilks, C., Zheng, S.C., Chen, F.Y., Charles, R., Solomon, B., Ling, J.P., Imada, E.L., Zhang, D., Joseph, L., Leek, J.T., et al. (2021). recount3: summaries and queries for large-scale RNA-seq expression and splicing. Genome Biol. *22*, 323. https://doi.org/10.1186/s13059-021-02533-6.
24. Dobin, A., Davis, C.A., Schlesinger, F., Drenkow, J., Zaleski, C., Jha, S., Batut, P., Chaisson, M., and Gingeras, T.R. (2013). STAR: ultrafast universal RNA-seq aligner. Bioinformatics *29*, 15–21. https://doi.org/10.1093/bioinformatics/bts635.
25. Ramírez, F., Ryan, D.P., Grüning, B., Bhardwaj, V., Kilpert, F., Richter, A.S., Heyne, S., Dündar, F., and Manke, T. (2016). deepTools2: a next generation web server for deep-sequencing data analysis. Nucleic Acids Res. *44*, W160–W165. https://doi.org/10.1093/nar/gkw257.
26. Dyer, S.C., Austine-Orimoloye, O., Azov, A.G., Barba, M., Barnes, I., Barrera-Enriquez, V.P., Becker, A., Bennett, R., Beracochea, M., Berry, A., et al. (2025). Ensembl 2025. Nucleic Acids Res. *53*, D948–D957. https://doi.org/10.1093/nar/gkae1071.
27. Taylor-Weiner, A., Aguet, F., Haradhvala, N.J., Gosai, S., Anand, S., Kim, J., Ardlie, K., Van Allen, E.M., and Getz, G. (2019). Scaling computational genomics to millions of individuals with GPUs. Genome Biol. *20*, 228. https://doi.org/10.1186/s13059-019-1836-7.
28. Barbeira, A.N., Bonazzola, R., Gamazon, E.R., Liang, Y., Park, Y., Ardlie, K., Lappalainen, T., and Im, H.K. (2020). Publicly available GWAS summary statistics, harmonized and imputed to GTEx v8' variant reference. Zenodo. https://doi.org/10.5281/zenodo.3629742.
29. Giambartolomei, C., Vukcevic, D., Schadt, E.E., Franke, L., Hingorani, A.D., Wallace, C., and Plagnol, V. (2014). Bayesian Test for Colocalisation between Pairs of Genetic Association Studies Using Summary Statistics. PLoS Genet. *10*, e1004383. https://doi.org/10.1371/journal.pgen.1004383.
30. Wang, G., Sarkar, A., Carbonetto, P., and Stephens, M. (2020). A simple new approach to variable selection in regression, with application to genetic fine mapping. J. R. Stat. Soc., Ser. B Stat. Methodol. *82*, 1273–1300. https://doi.org/10.1111/rssb.12388.
31. Kurki, M.I., Karjalainen, J., Palta, P., Sipilä, T.P., Kristiansson, K., Donner, K.M., Reeve, M.P., Laivuori, H., Aavikko, M., Kaunisto, M.A., et al. (2023). FinnGen provides genetic insights from a well-phenotyped isolated population. Nature *613*, 508–518. https://doi.org/10.1038/s41586-022-05473-8.
32. Vaquero-Garcia, J., Aicher, J.K., Jewell, S., Gazzara, M.R., Radens, C.M., Jha, A., Norton, S.S., Lahens, N.F., Grant, G.R., and Barash, Y. (2023). RNA splicing analysis using heterogeneous and large RNA-seq datasets. Nat. Commun. *14*, 1230. https://doi.org/10.1038/s41467-023-36585-y.
33. GTEx Consortium (2020). The GTEx Consortium atlas of genetic regulatory effects across human tissues. Science *369*, 1318–1330. https://doi.org/10.1126/science.aaz1776.
34. Weinstein, J.N., Collisson, E.A., Mills, G.B., Shaw, K.R.M., Ozenberger, B.A., Ellrott, K., Shmulevich, I., Sander, C., and Stuart, J.M. (2013). The Cancer Genome Atlas Pan-Cancer analysis project. Nat. Genet. *45*, 1113–1120. https://doi.org/10.1038/ng.2764.
35. Jansen, P.R., Watanabe, K., Stringer, S., Skene, N., Bryois, J., Hammerschlag, A.R., de Leeuw, C.A., Benjamins, J.S., Muñoz-Manchado, A.B., Nagel, M., et al. (2019). Genome-wide analysis of insomnia in 1,331,010 individuals identifies new risk loci and functional pathways. Nat. Genet. *51*, 394–403. https://doi.org/10.1038/s41588-018-0333-3.
36. Farshadi, E., van der Horst, G.T.J., and Chaves, I. (2020). Molecular Links between the Circadian Clock and the Cell Cycle. J. Mol. Biol. *432*, 3515–3524. https://doi.org/10.1016/j.jmb.2020.04.003.
37. Graham, S.E., Clarke, S.L., Wu, K.-H.H., Kanoni, S., Zajac, G.J.M., Ramdas, S., Surakka, I., Ntalla, I., Vedantam, S., Winkler, T.W., et al. (2021). The power of genetic diversity in genome-wide association studies of lipids. Nature *600*, 675–679. https://doi.org/10.1038/s41586-021-04064-3.
38. Suzuki, K., Hatzikotoulas, K., Southam, L., Taylor, H.J., Yin, X., Lorenz, K.M., Mandla, R., Huerta-Chagoya, A., Melloni, G.E.M., Kanoni, S., et al. (2024). Genetic drivers of heterogeneity in type 2 diabetes pathophysiology. Nature *627*, 347–357. https://doi.org/10.1038/s41586-024-07019-6.
39. Dorrell, C., Grompe, M.T., Pan, F.C., Zhong, Y., Canaday, P.S., Shultz, L.D., Greiner, D.L., Wright, C.V., Streeter, P.R., and Grompe, M. (2011). Isolation of mouse pancreatic alpha, beta, duct and acinar populations with cell surface markers. Mol. Cell. Endocrinol. *339*, 144–150. https://doi.org/10.1016/j.mce.2011.04.008.
40. Watanabe, K., Jansen, P.R., Savage, J.E., Nandakumar, P., Wang, X., Agee, M., Aslibekyan, S., Auton, A., Bell, R.K., Bryc, K., et al. (2022). Genome-wide meta-analysis of insomnia prioritizes genes associated with metabolic and psychiatric pathways. Nat. Genet. *54*, 1125–1132. https://doi.org/10.1038/s41588-022-01124-w.
41. Basu, K., Dey, A., and Kiran, M. (2023). Inefficient splicing of long non-coding RNAs is associated with higher transcript complexity in human and mouse. RNA Biol. *20*, 563–572. https://doi.org/10.1080/15476286.2023.2242649.
42. Yelmen, B., and Jay, F. (2023). An Overview of Deep Generative Models in Functional and Evolutionary Genomics. Annu. Rev. Biomed. Data Sci. *6*, 173–189. https://doi.org/10.1146/annurev-biodatasci-020722-115651.
43. Qiu, W., Dincer, A.B., Janizek, J.D., Celik, S., Pittet, M.J., Naxerova, K., and Lee, S.-I. (2024). Deep profiling of gene

expression across 18 human cancers. Nat. Biomed. Eng. *9*, 333–355. https://doi.org/10.1038/s41551-024-01290-8.

44. Way, G.P., and Greene, C.S. (2018). Extracting a biologically relevant latent space from cancer transcriptomes with variational autoencoders. Pac. Symp. Biocomput. Pac. Symp. Biocomput. *23*, 80–91.
45. Mattick, J.S., Amaral, P.P., Carninci, P., Carpenter, S., Chang, H.Y., Chen, L.-L., Chen, R., Dean, C., Dinger, M.E., Fitzgerald, K.A., et al. (2023). Long non-coding RNAs: definitions, functions, challenges and recommendations. Nat. Rev. Mol. Cell Biol. *24*, 430–447. https://doi.org/10.1038/s41580-022-00566-8.
46. Kerimov, N., Tambets, R., Hayhurst, J.D., Rahu, I., Kolberg, P., Raudvere, U., Kuzmin, I., Chowdhary, A., Vija, A., Teras, H.J., et al. (2023). eQTL Catalogue 2023: New datasets, X chromosome QTLs, and improved detection and visualisation of transcript-level QTLs. PLoS Genet. *19*, e1010932. https://doi.org/10.1371/journal.pgen.1010932.
47. Munro, D. (2026). PejLab/LaDDR: v1.0.0. Zenodo. https://doi.org/10.5281/zenodo.19921613.

The American Journal of Human Genetics, Volume *113*

Supplemental information

Data-driven RNA phenotyping captures genetically regulated dimensions of the transcriptome

Daniel Munro, Alexander Gusev, Abraham A. Palmer, and Pejman Mohammadi

Supplemental Information

In this file:
Figures S1-S11
Tables S1-S3 (descriptions)
Supplemental Methods

Supplemental Figures

Figure S1: Comparison of parameters and methodological variations.

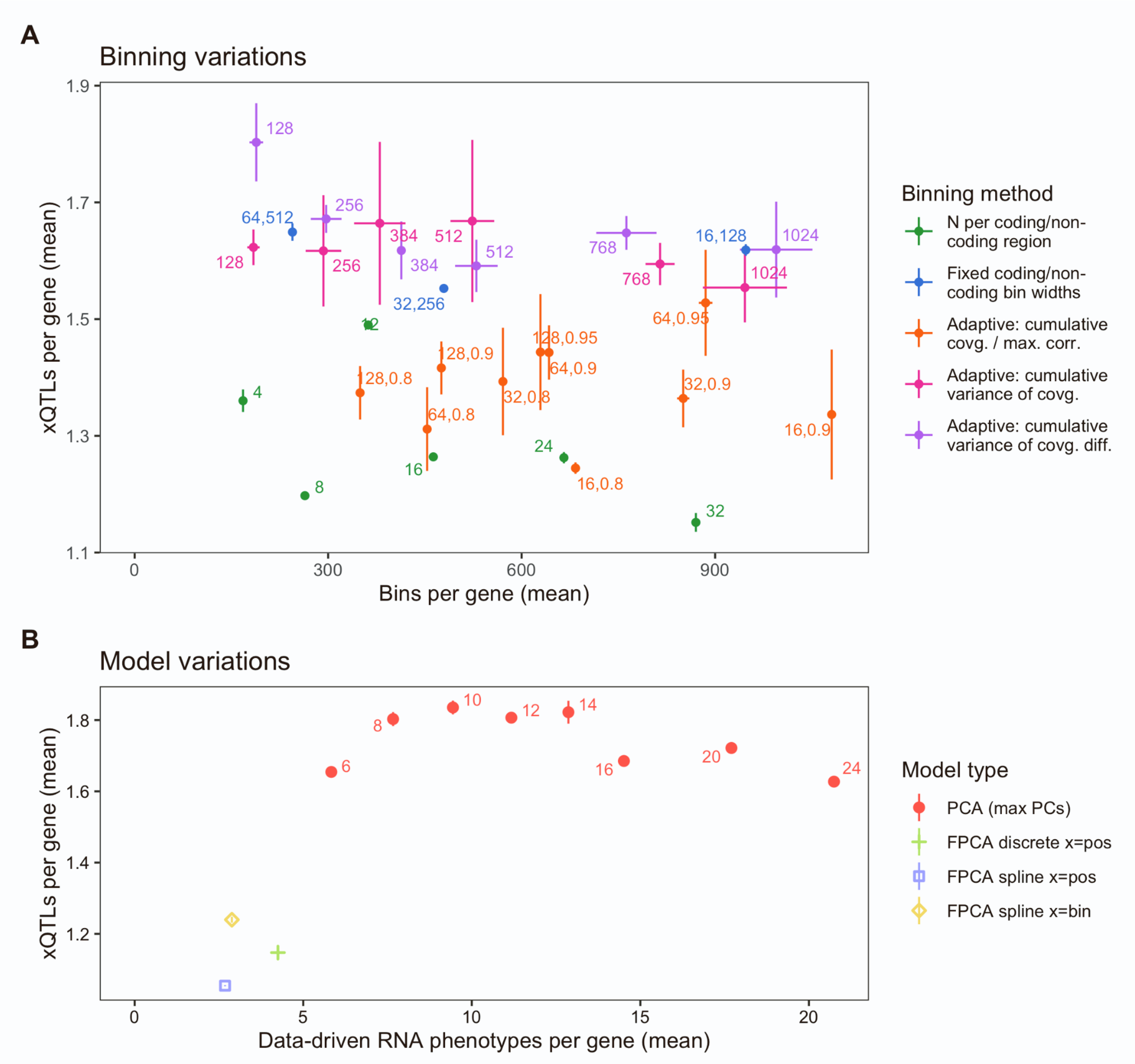

A Average number of bins per gene and conditionally independent DDP cis-xQTLs per gene for each binning method variation. Points and lines represent mean and standard deviation, respectively, across three trials for each method-parameter pair. Points are labeled with method-specific parameters as follows, following the order in the legend: number of bins per coding/noncoding region, bin widths for bins inside coding and noncoding regions, minimum cumulative mean coverage per bin and maximum correlation between adjacent bins, mean number of bins per gene, and mean number of bins per gene. **B** Average number of DDPs per gene and conditionally independent cis-xQTLs per gene resulting from each LaDDR gene model variation. Points for PCA models are labeled with the maximum number of PCs kept per gene.

Figure S2: Replication of GTEx LCL DDP xQTLs in Geuvadis

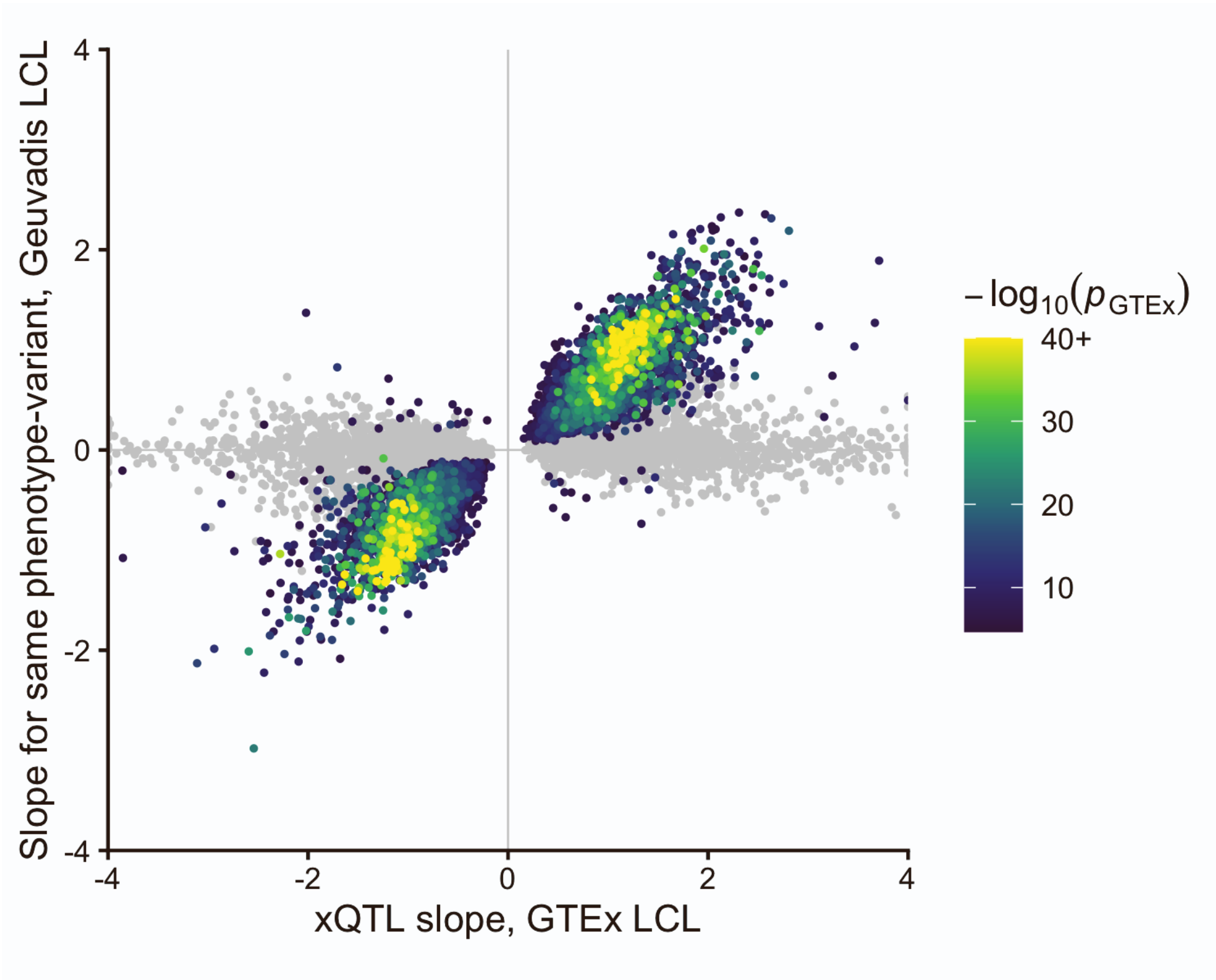

For each DDP xQTL lead variant for GTEx LCL tissue, effect size (slope) is shown on the x-axis, and slope for the same phenotype-variant pair in Geuvadis is shown on the y-axis. Points are colored by, and plotted in order of, nominal p-value in GTEx LCL, and points for phenotype-variant pairs that are not significant in Geuvadis (and thus do not have meaningful slopes) are gray.

Figure S3: xTWAS hits at different significance thresholds.

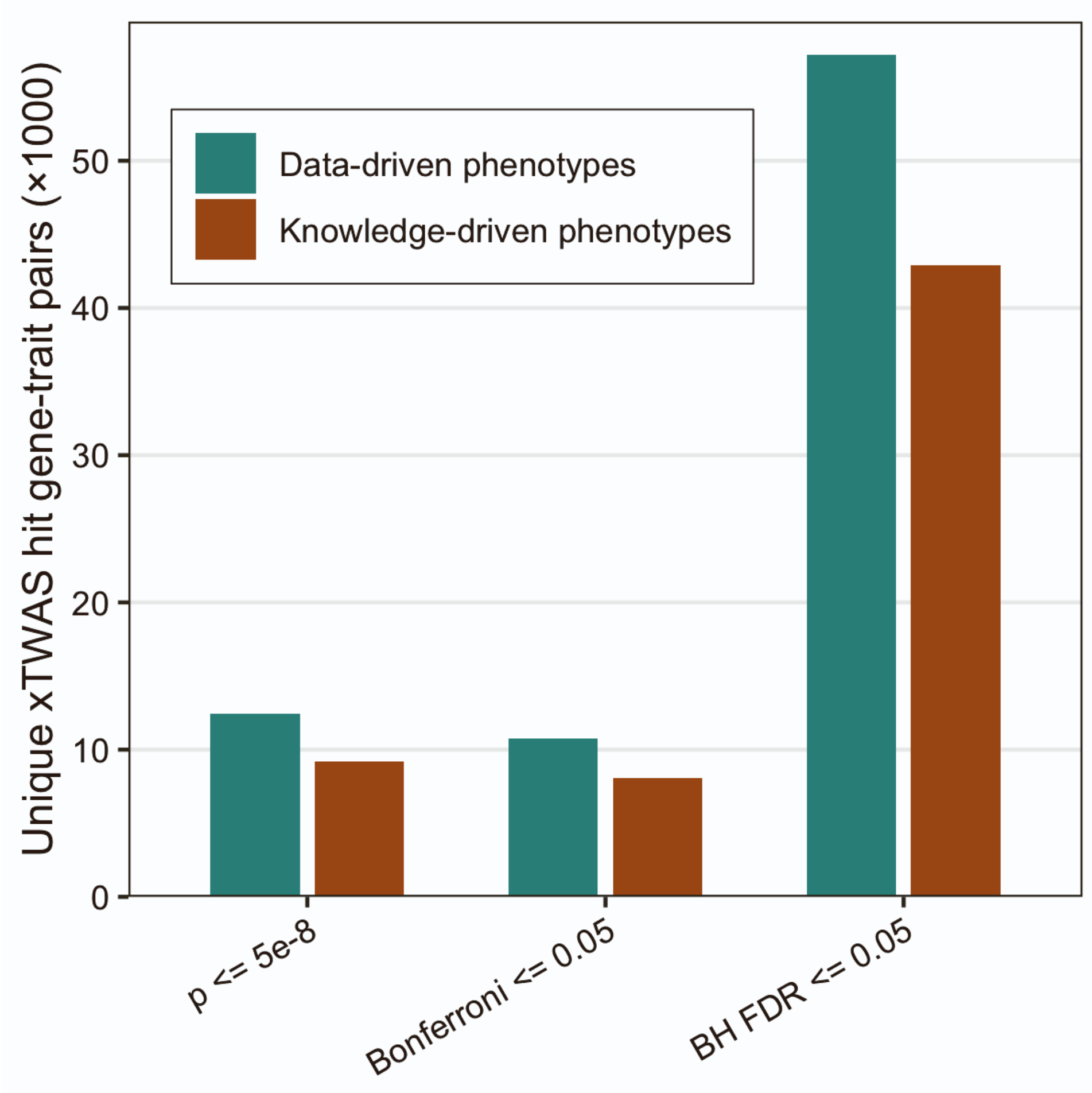

Number of unique gene-trait pairs with significant xTWAS associations in the Geuvadis dataset, when using various significance thresholds for DDP and KDP xTWAS results. “p <= 5e-8” is the fixed threshold used for the main results in this study. For “Bonferroni <= 0.05”, p-values are multiplied by the number of tests across all traits. For “BH FDR <= 0.05”, p-values are adjusted using the Benjamini Hochberg method to control false discovery rate across all traits.

Figure S4: New rDDP xQTLs when holding out specific knowledge-driven modalities.

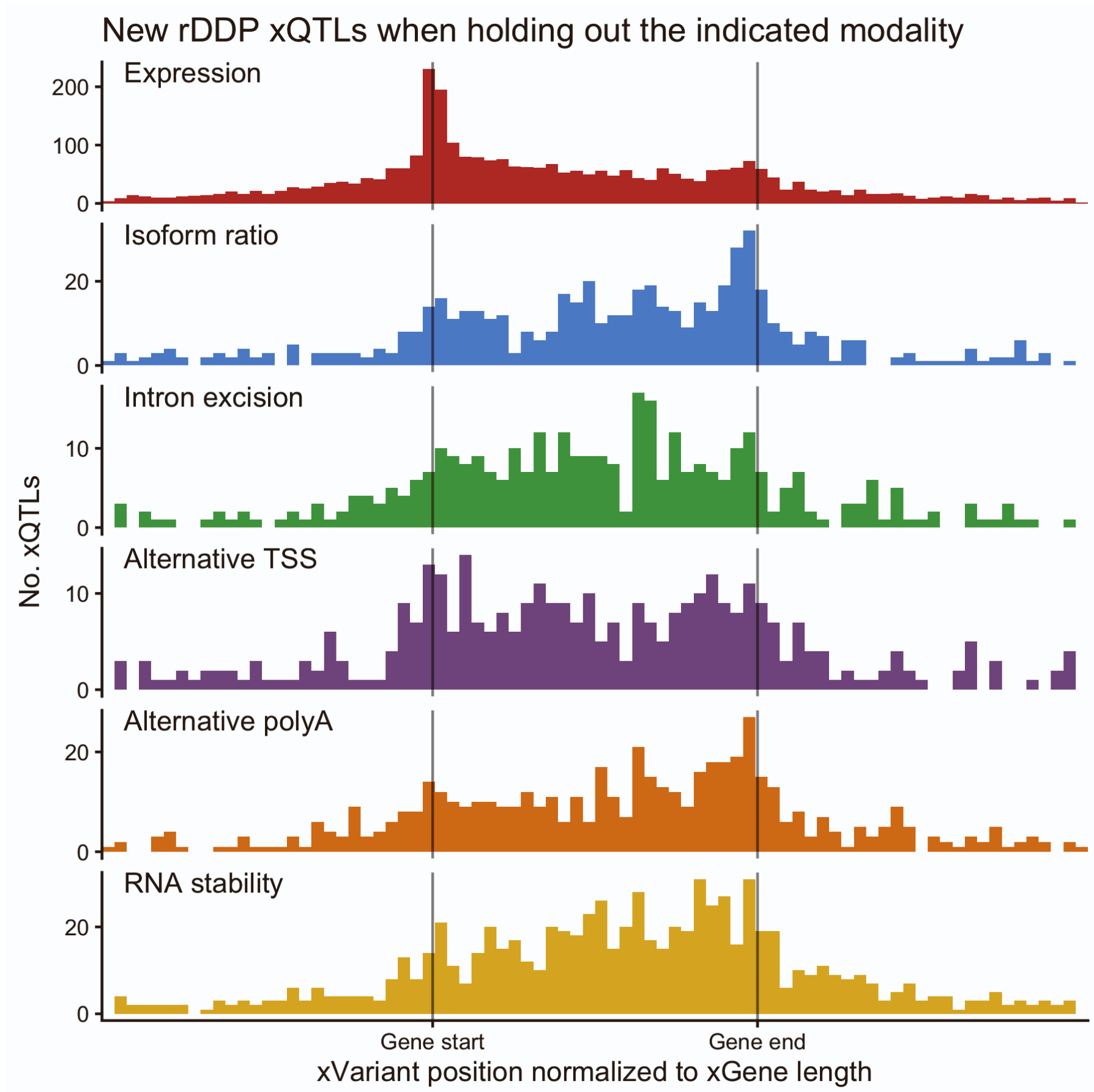

Histograms of rDDP xQTL locations relative to their xGene. The process of generating rDDPs and doing cross-modality mapping with KDPs and rDDPs was repeated six times, each time withholding one modality from the residualizing procedure and the xQTL mapping. Each xQTL shown is for an rDDP generated by holding out the indicated KDP modality, excluding those in genes that also had an rDDP xQTL in the non-held-out run. The genomic coordinates of each xGene and that gene's xQTLs were linearly transformed such that the gene starts and ends are aligned on the x-axis. xQTLs more than one gene length from the gene are not shown. Histograms are colored by modality.

Figure S5: Genotype-stratified RNA-seq coverage for xTWAS examples

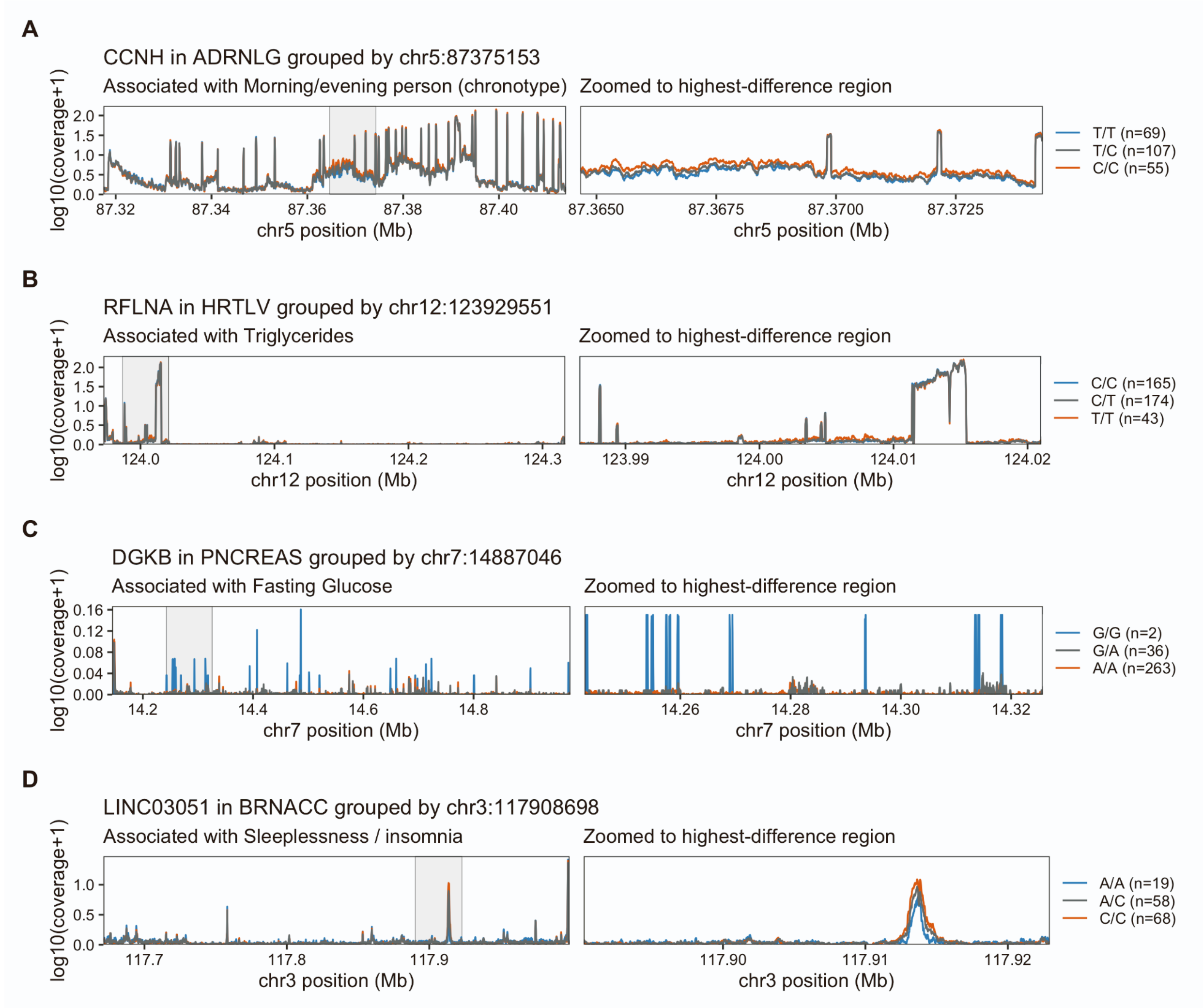

RNA-seq coverage plots for four selected genes with DDP and rDDP, but not KDP, xTWAS associations for a particular trait and tissue, as presented in the main text. Coverage is averaged across individuals with each genotype at the transcriptomic lead variant per xTWAS association. For each gene, the left panel shows the full gene range, with the 10% subrange with greatest absolute coverage difference between genotype groups highlighted with a gray box. Coverage in that subregion is expanded in the right panel.

Figure S6: Colocalization examples between xQTLs and FinnGen GWAS

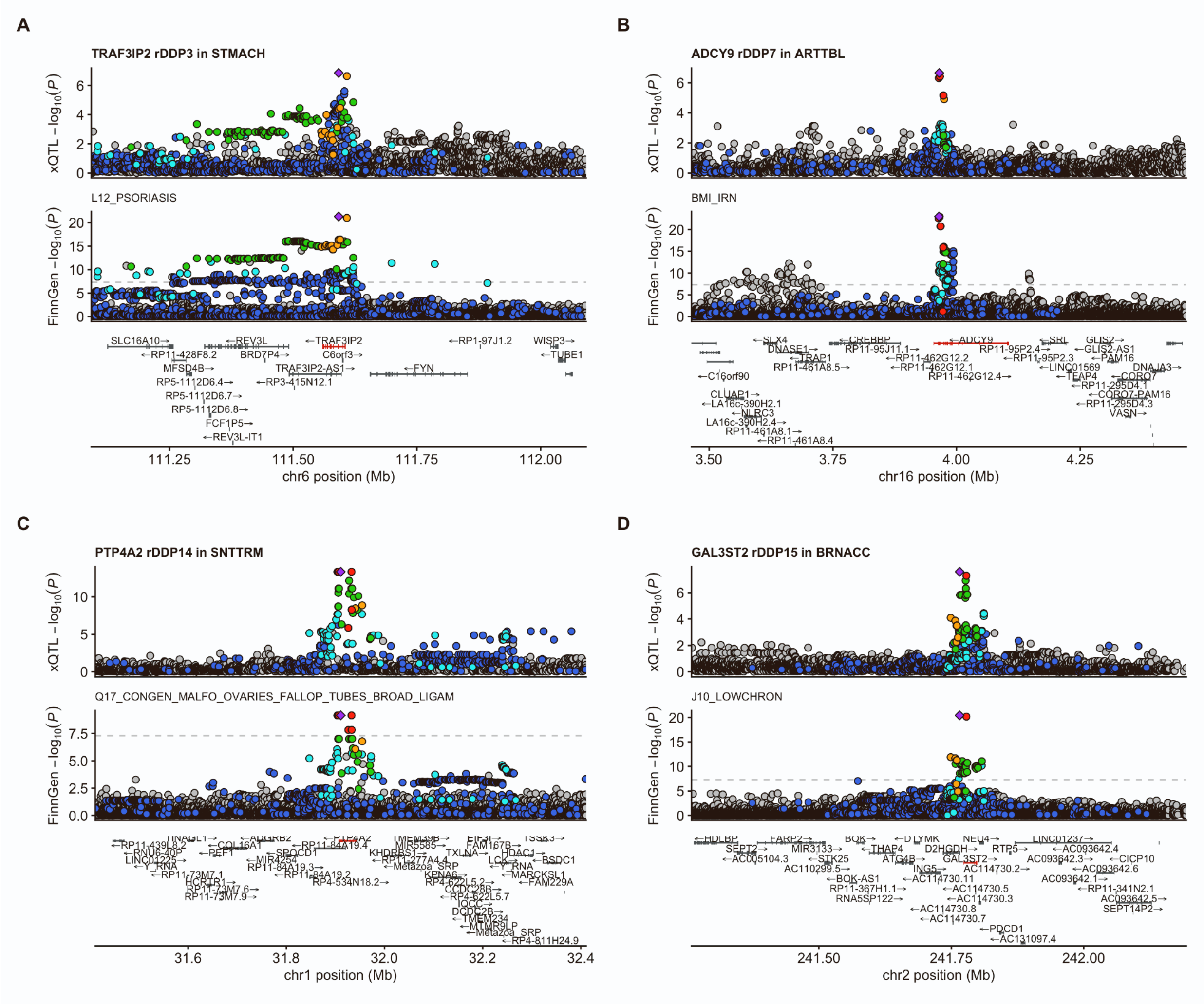

A-D Locuszoom-style plots for four selected rDDP xQTL colocalizations for gene-trait pairs that have no KDP colocalizations. The gene, phenotype, and tissue ID for the colocalizing rDDP xQTL are shown in the top panel label, and the FinnGen trait ID is shown in the bottom panel. The variant with the strongest evidence of colocalization is shown in both panels as a purple diamond and is used as the linkage disequilibrium (LD) reference for coloring the remaining points.

Figure S7: Additional colocalization to xQTL ratios

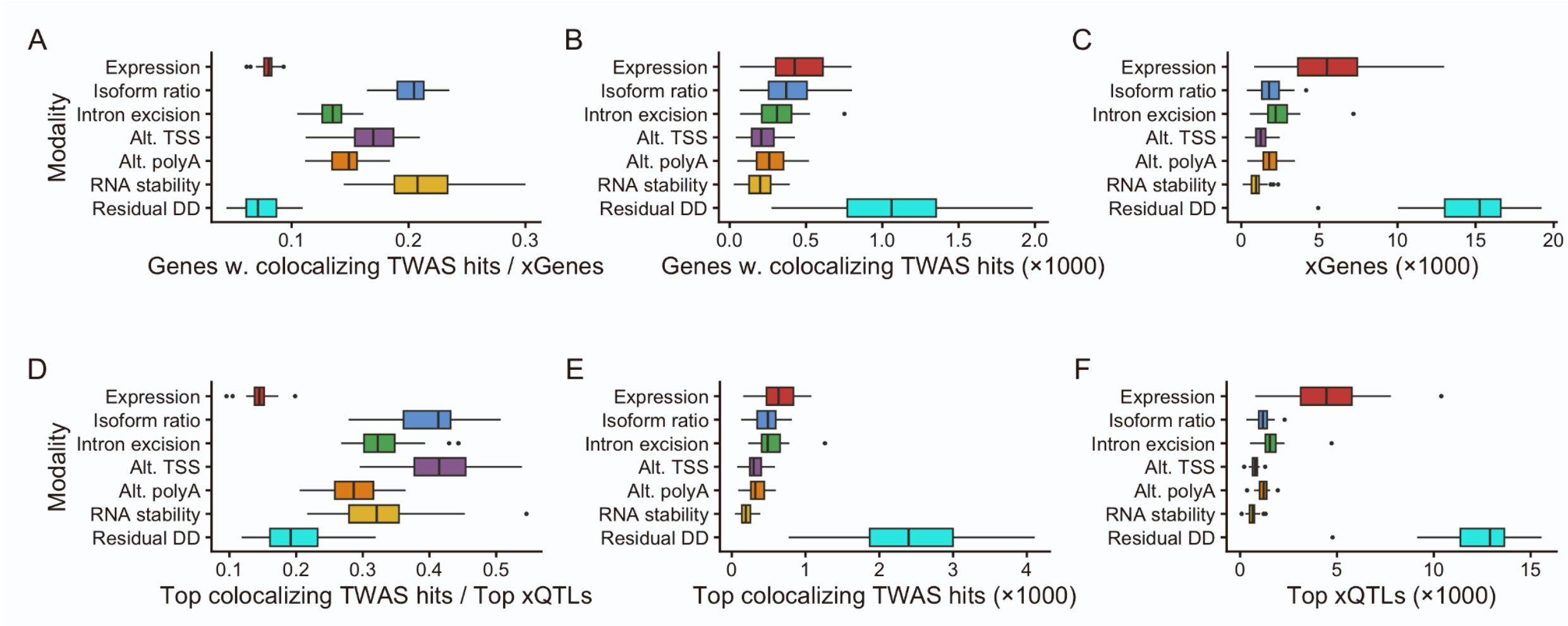

A For each modality, the number of genes with one or more colocalizing (COLOC.PP4 >0.8) xTWAS hits, divided by the number of genes with one or more xQTLs from hybrid cross-modality mapping, was computed for each GTEx tissue and shown as a boxplot. **B** The numerators for the ratios plotted in a are shown as boxplots. **C** The denominators for the ratios plotted in a are shown as boxplots. **D** For each tissue, the strongest colocalizing TWAS hit in terms of TWAS *p*-value per gene-trait pair was selected, and the number of those belonging to each modality was counted. Likewise, for each tissue, the strongest xQTL per gene from hybrid cross-modality mapping was selected, and the number of those belonging to each modality was counted. The ratio between these values are plotted as boxplots. **E** The numerators for the ratios plotted in d are shown as boxplots. **F** The denominators for the ratios plotted in d are shown as boxplots.

Figure S8: Percentage of xTWAS hits shared with nearby genes

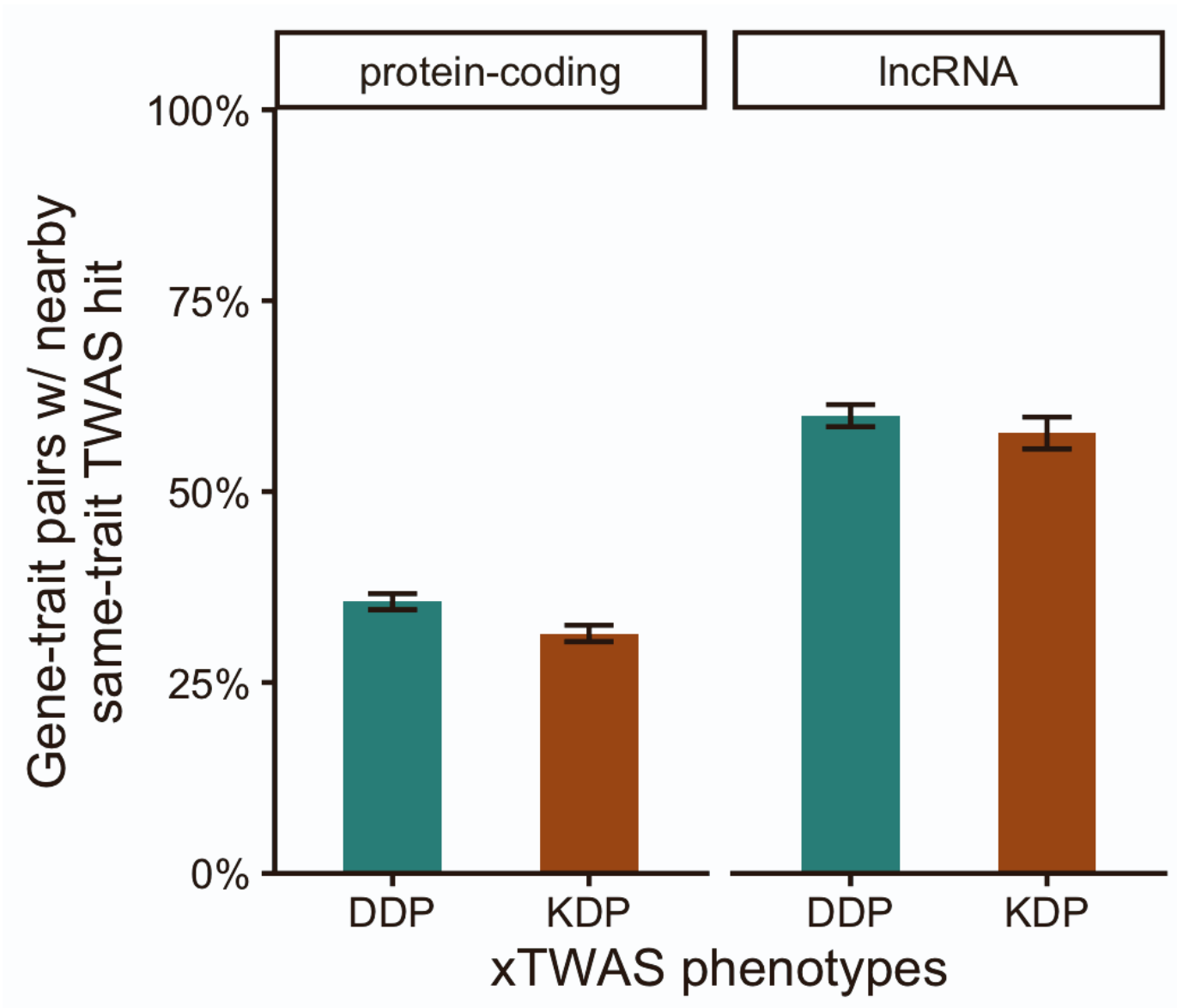

For each unique gene-trait pair with at least one DDP xTWAS hit, we examined whether there is a DDP xTWAS hit for the same trait for any nearby gene, defined as a gene whose range between transcription start and end overlaps with the first gene's range. We repeated this for KDP xTWAS hits and in both cases calculated the means separately for protein-coding and lncRNA genes. Error bars show 95% confidence intervals of each proportion. DDP = knowledge-driven phenotype, KDP = data-driven phenotype.

Figure S9: Performance of LaDDR models using different training data

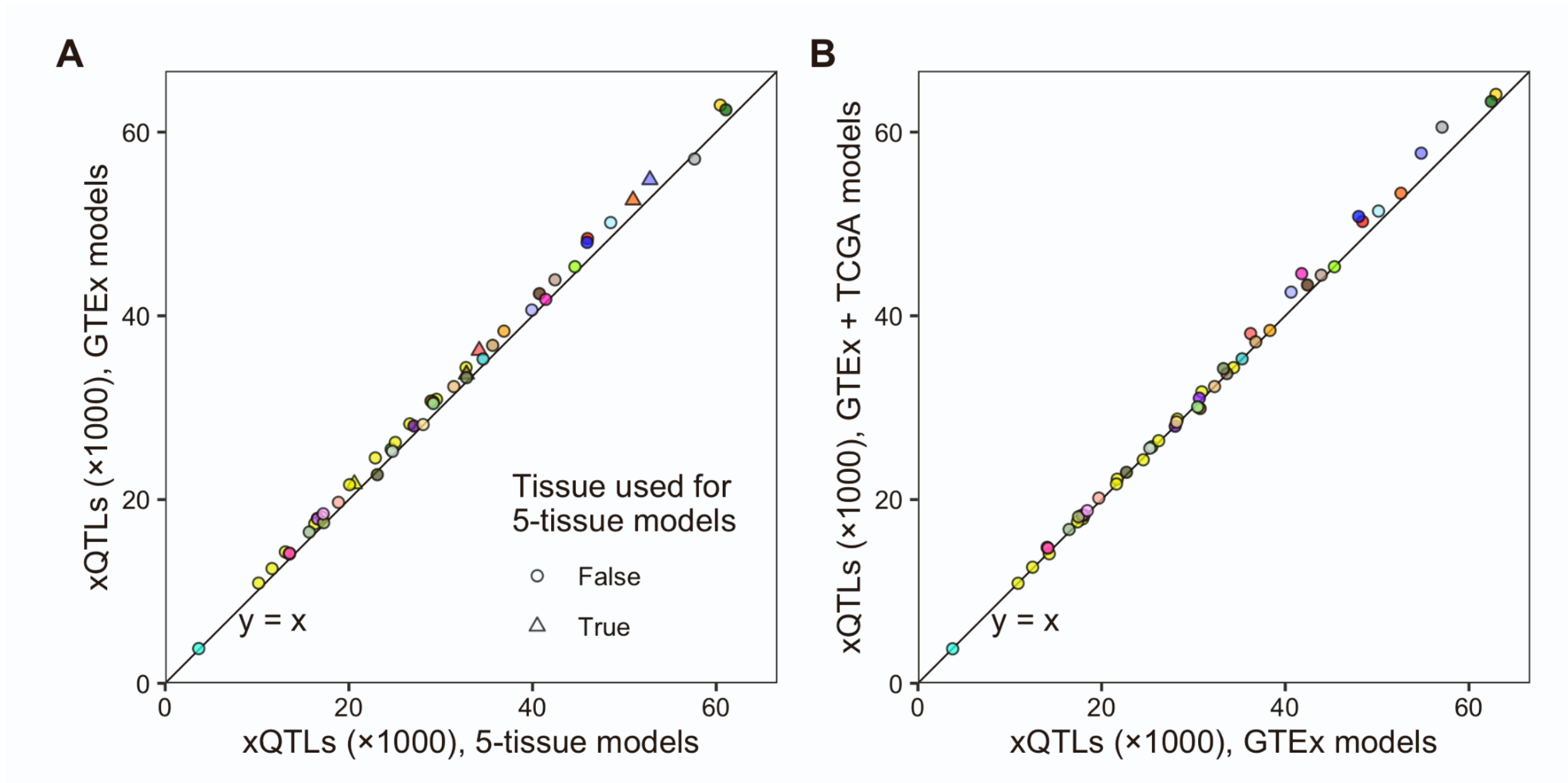

A Number of DDP xQTLs produced for each of 49 GTEx tissues when either all 54 GTEx tissues or only five GTEx tissues are used to fit the LaDDR gene models. **B** Number of DDP xQTLs produced for each of 49 GTEx tissues when either all 54 GTEx tissues plus 33 cancer types or only the 54 GTEx tissues are used to fit the LaDDR gene models.

Figure S10: xQTLs from pruned annotation simulation

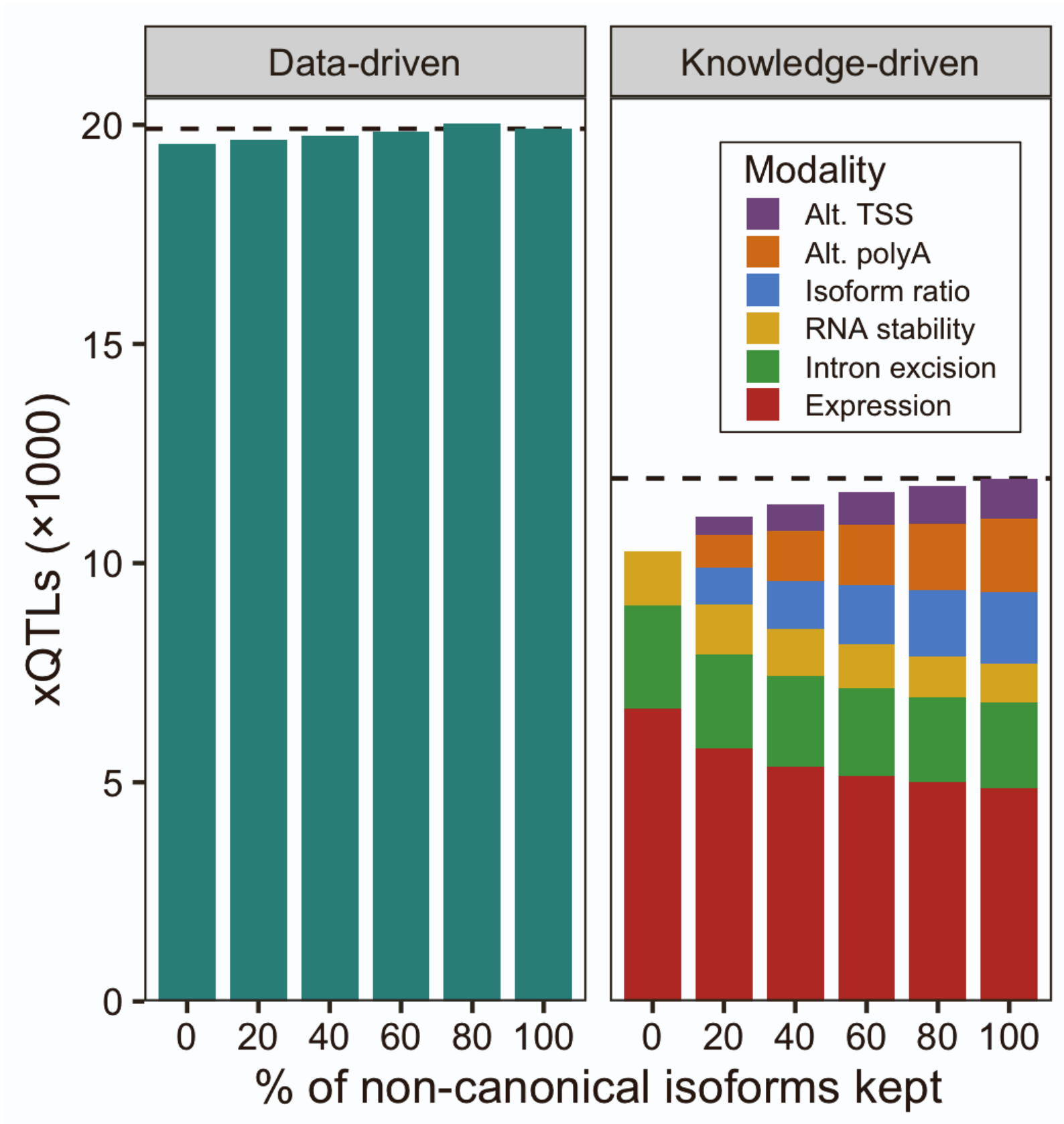

Number of xQTLs from DDPs (left) or KDPs (right) for the Brain - Frontal Cortex (BA9) tissue (N=175) after randomly subsampling non-canonical isoforms from the gene annotations used for phenotyping. Dashed lines mark the counts for 100% of isoforms for reference.

Figure S11: Simulated sequencing data variations

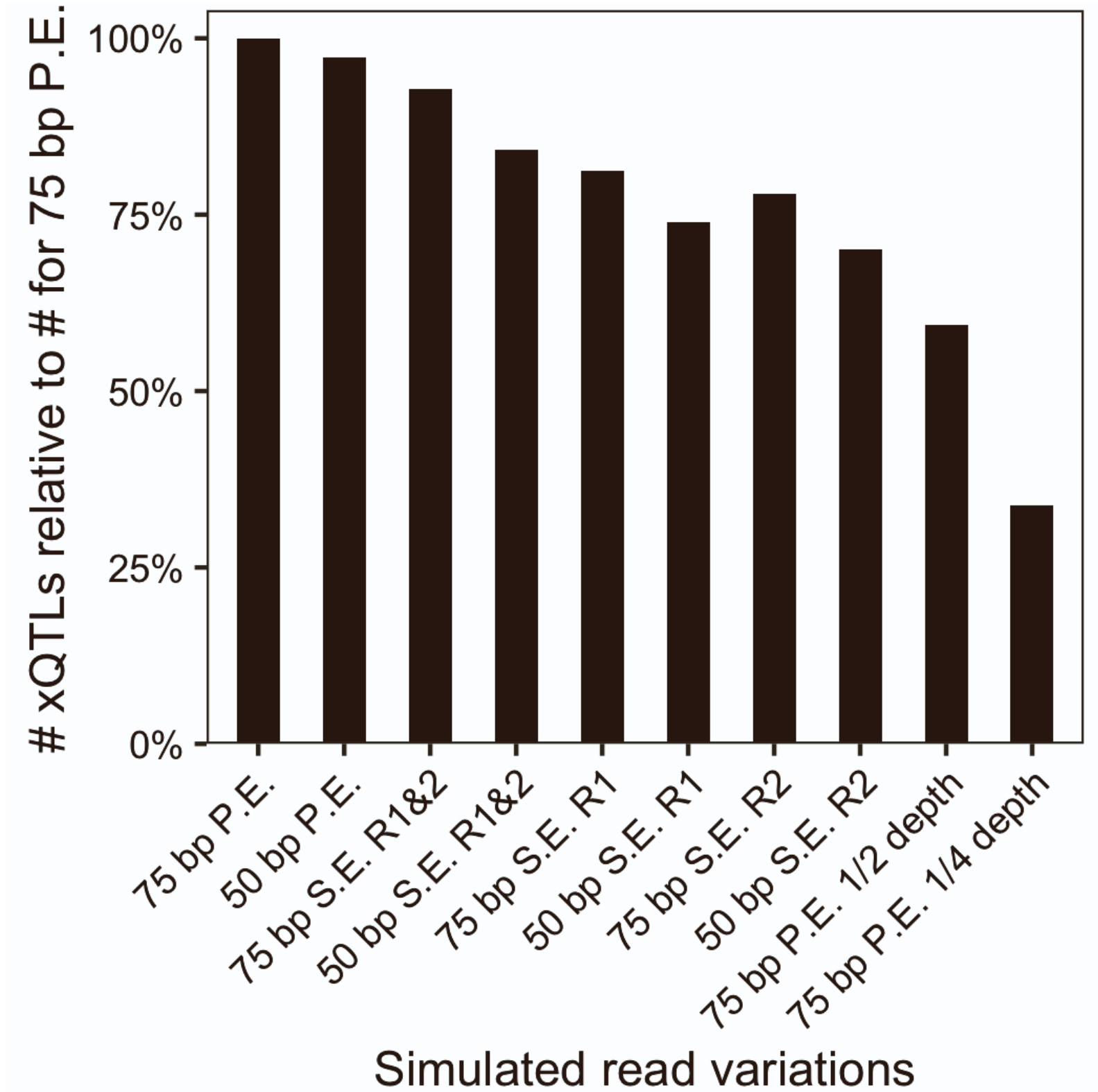

Number of DDP cis-xQTLs when using different variations of the same raw RNA-seq data. A random subsample of 100 Geuvadis samples (75 bp paired-end reads) were truncated to 50 bp, run with R1 and/or R2 reads run as single-end, or run on a subsample of reads equal to a fixed fraction (½ or ¼) of the original reads per sample. The original samples had an average of 30.1 million reads (SD 8.6 million).

Supplemental Tables

Table S1: xQTL count per tissue for DDP, hybrid (KDP + rDDP), and KDP phenotype sets

Table S2: xTWAS hit and gene counts per trait per tissue for DDP, rDDP, and KDP phenotype sets

Table S3: Colocalizations between KDP/rDDP xQTLs and FinnGen GWAS loci

Supplemental Methods

Comparison of binning methods and parameters

We compared the default binning method to four alternative methods, all currently available in the LaDDR software. Each method has one or more parameters that generally control the number and size of bins, so we tested the following ranges of parameter values for each method:

- **Fixed number of bins per gene subregion**:
    - Take the union of all exon regions for any isoform of a gene to get contiguous exonic regions, and split each region into a fixed number of equal-sized bins. Also split each region between adjacent exonic regions, as well as the 1kb upstream and downstream regions, into the same number of bins.
    - Range of parameter values compared: 4-32 bins per region.
- **Fixed bin widths**:
    - Using the same regions as above, split each coding (exonic) region into bins of a fixed width, and split each non-coding region into bins of a different fixed width chosen to be eight times larger than coding region bins.
    - Range of parameter values compared: 16/128 to 64/512 for coding/non-coding bin widths.
- **Data-driven binning based on cumulative coverage and coverage correlation**:
    - Start with each base as its own bin and merge bins in two stages:
        1. While any bin has mean cumulative coverage per sample below a threshold, merge it with whichever of its two neighbors has lower cumulative coverage, and repeat for the next lowest-coverage bin.
        2. Measure correlation between neighboring bins as Pearson correlation of $\log_2$(norm. coverage + 1) across samples. Normalized coverage means, for each sample, the fraction of cumulative coverage across the gene that is within the bin. While any neighboring bins have correlation above the threshold, merge them and repeat.
    - Range of parameter values compared: 16-128 minimum cumulative mean coverage per bin, 0.8-0.95 maximum correlation between adjacent bins.
- **Data-driven binning based on cumulative variance of coverage**:

    - Determine the cumulative variance threshold: Get variance of $\log_2$(coverage + 8) at each base. Get sum of variance across each gene (for a sample of 128 genes for efficiency), get mean sum per gene, and divide by desired number of bins per gene to get sum of variance per bin. Spikes of variance can skew the resulting number of bins per gene away from the intended target, so the following procedure is performed once on the sample of 128 genes and the threshold is adjusted to accurately produce the desired number of bins per gene. Binning is then performed for all genes.
    - For each gene, start with the first base and extend the bin until the cumulative-variance-per-bin threshold is reached. Repeat until reaching the end of the gene's range.
    - Range of parameter values compared: 128-1024 mean bins per gene.
- **Data-driven binning based on cumulative variance of coverage diffs (the default)**:
    - Identical to the previous method, but compute the differences in adjacent values for $\log_2$(coverage + 8) at each base, and calculate variance of those (rather than on $\log_2$(coverage + 8) directly) to determine the cumulative variance threshold and the bin boundaries.
    - Range of parameter values compared: 128-1024 mean bins per gene.

For each set of parameter values for each method, we ran the full phenotyping procedure with otherwise default parameters (e.g., PCA models with a maximum of 16 PCs per gene) on the Geuvadis dataset. We then performed cis-QTL mapping on the DDPs to use independent cis-QTLs as a metric for each binning's ability to capture cis-regulatory variation. For efficiency, we computed DDPs and cis-QTLs only for chr7 (932 genes).

Comparison of model types and parameters

We compared the use of PCA to functional PCA as the LaDDR phenotyping model. While basic PCA uses the normalized binned coverage values as an unordered set of input variables, functional PCA can use the relative position of the bins along the gene sequence as additional information. We compared the following variants of PCA and FPCA:

- **PCA**: For each gene, keep enough PCs to capture at least 80% variance explained, then clipped to a fixed maximum number of PCs. Range of parameter values compared: 6-24 maximum PCs kept.
- **Discretized FPCA**: Use the `scikit-fda` Python package, providing the binned coverage features in order.
- **FPCA with spline basis using bin coordinates**: Use a 4th-order BSpline basis, using ordered bin numbers as domain coordinates.
- **FPCA with spline basis using equal bin spacing**: Use a 4th-order BSpline basis, using the middle coordinate of each bin as domain coordinates.

As with the binning comparison, we generated DDPs for the Geuvadis dataset using each of these variants with otherwise default parameters (e.g., data-driven binning based on cumulative

variance of coverage diffs with average bins per gene of 256), and then mapped cis-QTLs for chr7 genes.
